# Effect of Hexagonal Boron Nitride (h-BN) Content on the Mechanical and Tribological Properties of Fused Deposition Modeling (FDM)-Printed PETG Composites

**DOI:** 10.3390/polym18192365

**Published:** 2026-09-28

**Authors:** Ömer Barışkan Yasan

**Affiliations:** Department of Mechanical Engineering, Erciyes University, Kayseri 38280, Turkey; omeryasan@erciyes.edu.tr

**Keywords:** PETG, hexagonal boron nitride, fused deposition modeling, additive manufacturing, solid lubricant, tribology, wear, coefficient of friction, nanocomposite

## Abstract

PETG composite filaments containing 1 wt%, 3 wt%, and 5 wt% hexagonal boron nitride (h-BN) were produced. The produced filaments were transformed into test specimens using the fused deposition modeling (FDM) technique. The tensile, compression, and wear behaviors of the composite polymer test specimens produced by the FDM method were investigated. The wear characteristic was measured using a ball-on-flat tribometer under normal loads of 5, 10, and 15 N. The worn surfaces were characterized by profilometer, scanning electron microscopy (SEM), and energy-dispersive X-ray spectroscopy (EDX). PETG/1hBN showed the lowest coefficient of friction (COF) at all tested loads and provided a 40–46% reduction compared to neat PETG. In addition, it was observed to have the wear track with the lowest depth and the smoothest surface. Furthermore, the highest compressive yield strength among the investigated compositions was reached in these specimens. For specimens containing higher h-BN contents, a proportional improvement could not be achieved, and this was interpreted as being due to the agglomeration of the filler material reducing its effectiveness in the transfer film layer. These results show that the addition of a low amount of h-BN to PETG is an effective and easily processable method for improving the wear resistance of the PETG matrix.

## 1. Introduction

Additive manufacturing (AM), specifically fused deposition modeling (FDM), has in recent years evolved beyond being merely a rapid prototyping technique and has become a viable method for the production of thermoplastic components intended for end users [1,2]. However, the production of end-use parts by FDM also brings along some macro- and micro-scale defects such as voids, weak interlayer adhesion, and anisotropic microstructures. This generally reduces the mechanical and tribological performance of printed parts compared to their injection-molded counterparts [1,3]. Since many components produced by FDM (gears, bushings, and sliding bearings, etc.) are ultimately produced for light-duty conditions, understanding and improving their sliding wear behavior has become an active research area [4,5]. PETG polymer was selected due to its good printability, ease of processing for the FDM method, and widespread use in additive manufacturing applications. Although it does not have high hardness, it was selected in this study not to increase hardness, but rather to investigate the effect of h-BN particles on the tribological performance of the polymer. The composites that were the subject of this study were aimed at enabling the easy production of friction elements operating under a low load range by additive manufacturing. It has been shown that process parameters such as layer thickness, printing speed and angle, infill density, and post-printing treatments significantly affect the coefficient of friction (COF) and specific wear rate in parts produced by FDM [6,7]. On the other hand, due to reasons such as thermally induced oxidations during the printing process, the surface chemistry of the polymer may change, and this makes the tribological characterization of AM parts more complex compared to those produced by conventional methods [8].

Polyethylene terephthalate glycol (PETG) has gained increasing popularity compared with polylactic acid (PLA) and acrylonitrile butadiene styrene (ABS), which can be printed by FDM, due to its strength, ductility, chemical resistance, low thermal shrinkage, and ease of printability [9,10]. There are numerous studies on the effects of printing parameters of PETG parts produced by FDM, such as nozzle temperature, infill density, layer height, and printing speed, on tensile and flexural properties [9,10,11]. Nevertheless, carbon fiber and nanofiller reinforcements have been investigated for the purpose of improving mechanical performance. Despite all these studies, the wear resistance of PETG has received relatively less attention. A recent study by Vaught and Polycarpou [12] revealed an unexpected result. FDM-produced parts have defects such as voids and weak bonds due to the layered manufacturing techniques, and therefore they are expected to exhibit poorer performance compared to parts produced by conventional methods. However, in this study, PETG exhibited a 10 times better wear resistance in dry sliding wear compared to PETG produced by compression molding. This effect was attributed to surface oxidation occurring during printing and the formation of a transfer film layer. Nevertheless, compared with engineering polymers such as PEEK (polyether ether ketone) or PTFE (polytetrafluoroethylene), it corresponds to a wear rate that is 10–100 times higher. In subsequent studies, this gap has been attempted to be closed by adjusting the infill density by reinforcing FDM-printed PETG with carbon fiber [13], by tuning the infill density and post-printing annealing of carbon fiber–PETG composites [14], by using short carbon fibers combined with organically modified montmorillonite [15], and in recent studies, by using low amounts of graphene [16,17]. These studies confirm that the coefficient of friction and specific wear values can be reduced with the addition of suitable filler materials in parts printed by FDM, and similar trends are also observed for other FDM thermoplastics such as ABS and PLA [18].

The improvement of polymer tribology by particulate filler materials is generally explained by the formation mechanism of the transfer film layer. Filler materials that support the formation of a thin, stable, and well-adhered transfer film layer on the metallic surface reduce surface wear and stabilize the sliding contact. In contrast, transfer films that are weakly bonded to the surface and form irregularly are associated with high wear [19,20,21]. This framework, which has been developed through the evaluation of various compositions of PTFE- and PEEK-based materials [22,23], has guided the ongoing investigation of filler materials that exhibit both low interlayer shear resistance in the layers formed on the friction surfaces and strong interfacial adhesion to the polymer matrix.

Hexagonal boron nitride (h-BN) is exactly such a filler material. Structurally, it is similar to graphite and consists of covalently bonded B-N layers. These layers are bonded to each other by weak Van der Waals secondary bonds. This allows the layers to slide relative to each other under contact stress and to align parallel to the direction of loading, forming a low-friction, layered film [24]. Unlike graphite and MoS2, it provides high in-plane thermal conductivity. Along with the lubricating effect of h-BN, its in-plane thermal conductivity [25,26] is also expected to affect the thermal behavior of the printing process. This may alter the heat distribution between layers and the local cooling rates during printing. It is known that this affects the interlayer adhesion of parts printed by FDM and, consequently, their mechanical and tribological properties. This is therefore considered an additional factor in the additive manufacturing of PETG/h-BN composites [25,26]. It exhibits excellent thermal and chemical stability up to approximately 900 °C [24] and provides electrical insulation. Particularly in consumer products and biomedical applications, boron nitride nanomaterials have been reported in the literature to be highly biocompatible and to exhibit low cytotoxicity, a very important property [27,28]. Compared with graphite and MoS2, these properties, together with its clean character, have made h-BN an attractive solid lubricant additive for oils and greases [29,30,31] and a filler material used in polymer matrices.

Numerous studies have clearly demonstrated that h-BN added at low concentrations improves the tribological performance of polymers. Joshi et al. [32] found that the specific wear rate of polyaryl ether ketone (PAEK) decreased by 22 times with the addition of only 0.5 wt% h-BN, while with increasing content, the transfer film quality decreased and wear increased as a result of agglomeration. In a similar study, De Oliveria et al. [33] reported an improvement of 56–70% in the wear rate of polyimide/h-BN nanocomposites both at room temperature and at high temperature, and attributed this effect to the formation of a tribofilm caused by h-BN and the reduction in shear stress at the sliding interface. Similar effects have also been reported for h-BN-filled polyamide 66 [34], polyamide 6/glass fiber [35], and neat nylon 6 [36] at 1–4 wt% concentrations. At loadings above this ratio, the tribological gains initially obtained were reversed due to particle agglomeration, interfacial roughness, and decreased hardness [35,36]. Similar and non-monotonic effects have been reported in short glass fiber-reinforced h-BN/PAEK composites [37]. h-BN has been used as a secondary solid lubricant in PAEK composites [38], epoxy resins [39], and PTFE matrix systems [40]. In addition, h-BN has been combined with other fillers such as reduced graphene oxide and nano-graphite, or used together with polydopamine surface-modified fillers, in order to improve the mechanical and tribological performance of polyimide and epoxy matrices to a higher level, and more successful results have been obtained [41,42,43]. Unlike tribology, h-BN is also widely used at low filler contents to increase thermal conductivity [44]. In addition, it is also being investigated as an environmentally friendly and non-residue-forming lubricant additive for water-based systems [45].

Despite the various comprehensive studies conducted on h-BN-reinforced polymers processed using conventional methods such as press molding, injection molding, hot pressing, etc., studies in which h-BN reinforcement is applied using the FDM method are quite limited. To date, the closest study related to this subject was conducted by Ralls et al. [46], in which Al_2_O_3_ and h-BN were sprayed in situ onto ABS parts printed by FDM. Although a decrease in strength values occurred due to the porous structure caused by the filler material, significant improvements in frictional resistance were observed under severe loading conditions. To the best of our knowledge, no study to date has systematically investigated the effect of h-BN content on the ball-on-flat tribological behavior, changes in surface roughness, and worn surface morphology of FDM-printed PETG. This combination is particularly relevant since it has previously been reported that the wear behavior of FDM-PETG is sensitive to changes in surface chemistry occurring during processing [12], and it has been documented that the optimum filler content for the wear behavior of h-BN-filled composites is achieved at a specific level rather than exhibiting a monotonically increasing behavior depending on the filler ratio [32,35,36].

In this study, PETG/h-BN composite filaments containing 1%, 3%, and 5% h-BN by weight were produced. These filaments were converted into ball-on-flat test specimens using the FDM method. The coefficient of friction and wear track morphology were tested under three different normal loads (5 N, 10 N, 15 N). The worn surfaces were characterized in more detail using contact profilometry, scanning electron microscopy (SEM), and energy-dispersive X-ray spectroscopy (EDX). For the same composites, tensile and compression specimens were also printed using the FDM method and mechanical characterization was performed. The main objective of this study is to determine the optimum h-BN ratio for FDM-produced PETG specimens in terms of friction, wear resistance, and mechanical performance, and to clarify the wear mechanisms that occur by correlating them with surface composition analyses.

## 2. Materials and Methods

### 2.1. Materials

The primary matrix polymer used was polyethylene terephthalate glycol (PETG), commercial grade CPT-3912 (ZheJiang KaiPuQi New Materials Technology Co., Ltd., Haining, China). According to the manufacturer’s datasheet, the product has a density of 1.29 g/cm^3^, an intrinsic viscosity of 0.760 ± 0.020 dL/g, a glass transition temperature of 80 °C, and a peak melting temperature of 200.0 ± 5.0 °C. The mechanical properties specified by the manufacturer are as follows: tensile yield strength of 53.7 MPa, melting temperature of 200 °C, elongation at break of 30%, and tensile elastic modulus of 2220 MPa. As the solid lubricant filler material, h-BN powder with an average particle size of 790 nm from the Molchem brand was used.

### 2.2. Filament Production

PETG/h-BN composite filaments were prepared by a two-stage melt extrusion process, as schematically illustrated in Figure 1. Before both extrusion processes, the materials were dried at 60 °C for 24 h to minimize the porosity and extrusion inconsistencies that may occur during production due to moisture. In the first stage, a mixture containing 10 wt% h-BN was prepared using a co-rotating twin-screw extruder (Gülnar Makina, Kayseri, Türkiye, L/D: 48, D: 16 mm) (Figure 2a) at a screw speed of 200 rpm and with a temperature profile of 230, 235, 235, 220, and 215 °C from the feed hopper to the die. The extrudate coming out of the twin-screw extruder was granulated using a crusher.

In the second stage, the PETG composite masterbatch containing 10 wt% h-BN was dosed using a SAVSAN (Savsan Makina A.Ş., Kayseri, Türkiye) brand single-screw extruder (Figure 2b,c) with a screw diameter of 30 mm and an L/D ratio of 24, equipped with a laser diameter control unit and a winding unit, and filaments containing 1 wt%, 3 wt%, and 5 wt% h-BN were produced. During this extrusion process, the screw rotation speed was set at 36 rpm and the feed-to-die temperature profile was determined as 180, 190, 200, and 200 °C. The obtained filaments were measured as 1.75 mm ± 0.05. In order to evaluate the mechanical and tribological performance of the filler material ratio, neat PETG and filaments containing 1 wt%, 3 wt%, and 5 wt% were produced. The sample coding, for example, PETG/3hBN, represents a composite filament containing 3 wt% h-BN.

### 2.3. Fused Filament Fabrication Test Specimens

Neat PETG and composite filaments containing h-BN (1%, 3%, and 5% by weight) were printed using a Bambu Lab P1S Combo printer (Bambu Lab, Shenzhen, China) to produce the test specimens as shown in Figure 2. During printing, a 0.4 mm steel nozzle was used and the layer thickness was set to 0.2 mm. In order to observe the effect of the material on the printed specimens, the specimens were produced with a 100% infill ratio, as in plastic injection molding. A textured PEI build plate was used during printing, and the printing parameters are given in Table 1. These printing parameters are compatible with those used to optimize the mechanical properties of PETG material in previous studies [9,10]. The layer thickness and raster angle values were selected by examining their effects on tribological behavior [6,7].

Three different specimen geometries were selected and designed in accordance with the test standards as shown in Figure 3.

- Tensile specimens were produced in a dog-bone structure with a total length of 63.5 mm, a thickness of 2 mm, and a width of 10 mm, in accordance with the ASTM D638/Type V standard [47].
- Compression specimens were designed with a diameter of 10 mm and a length of 20 mm, taking into account the aspect ratio specified by the ASTM D695 standard, and cylindrical specimens were produced [48].
- Ball-on-flat wear specimens were produced with dimensions of 30 × 30 × 5 mm in accordance with the ASTM G133 standard [49] as shown in Figure 4.

All specimens were printed with a 100% infill ratio, allowing the composites to be evaluated in terms of bulk material properties without the void effect caused by the infill patterns.

### 2.4. Mechanical Tests (Tensile and Compression)

The average tensile strength and elastic modulus of the specimen groups produced, four different parameter groups including one neat PETG, and the compressive yield stress were determined by performing tensile and compression tests. These tests were performed to support the tribological findings and to facilitate their interpretation. In particular, they help to quantitatively determine the quality of the filler material–matrix interfacial bonding, which provides useful implications for the tribological findings.

Tensile and compression tests were performed using MTS Criterion Model 43 (MTS Systems Corporation, Eden Prairie, MN, USA), which is a double-column electromechanical universal testing machine. The device has a 50 kN load capacity, a speed range of 0.005–2000 mm/min, and a digital closed-loop control unit operating at 1000 Hz. All specimens were fixed using mechanical grips. Prior to all tests, the specimens were conditioned at room temperature for 12 h.

For the purpose of evaluating dimensional stability after FDM printing, measurements were taken in the x, y, and z directions from three different unpolished and unworn specimens for all compositions and compared with the dimensions in the CAD design. As summarized in Table 2, the deviation in the nominal dimensions remained below 1% for all compositions. This confirms the dimensional accuracy and repeatability of all compositions during the printing process.

Tensile tests (Figure 4) were performed using specimens designed in accordance with the ASTM D638-Type V standard, at a tensile speed of 0.2 mm/min and at a room temperature of 23 °C. The reason for using this low speed is to provide quasi-static conditions during the tensile testing of polymeric nanocomposites. The strain amount was calculated from the displacement of the crosshead. The test continued until each specimen fractured. All tests were repeated three times to ensure repeatability. The data obtained from these tests were used to determine the ultimate tensile strength, yield strength, and elastic modulus values.

Compression tests (Figure 5) were also performed using the same device in accordance with the ASTM D695 standard. The crosshead speed of 0.2 mm/min, which was used in the tensile tests, was also used here, and the tests were performed under the same environmental conditions. Again, each compression test was repeated three times.

### 2.5. Wear Tests

The wear specimens were tested under dry sliding conditions in a reciprocating configuration using a UTS Tribolog (UTS Scientific Instruments, Trabzon, Türkiye) (Figure 6) at room temperatures of 20–26 °C. The tests were conducted in accordance with the ASTM G133 standard [49]. All tests were performed at a frequency of 5 Hz with a stroke length of 10 mm over a distance of 250 m, using different normal loads of 5, 10, and 15 N. For each load combination, the tests were repeated three times and the average COF values were determined accordingly. Representative average curves were plotted accordingly. Standard deviation data for the COF values were indicated on the bar graphs. A 6 mm diameter 100Cr6 steel ball was used as the counter surface for the tests.

Prior to the tests, the tested surfaces of all specimens were polished in order to prevent inconsistencies that may be caused by the layered printing surfaces on the friction surface. In addition, all surfaces were cleaned by washing with pure water after polishing to prevent possible contaminants from affecting the measurements during the tests.

The coefficient of friction (COF) was obtained from the friction force data recorded in real time by a load cell integrated into the tribometer. This test configuration is also a method adopted in previous studies for investigating the wear behavior of polymer composites [50,51].

### 2.6. Surface Characterization

#### 2.6.1. Contact Profilometry

The topography of the wear tracks formed on the surface as a result of the wear tests was characterized using a contact-type, 2D surface profilometer (DektakXT, Bruker Corporation, Billerica, MA, USA). The measurements were obtained by scanning the specimen with a precise stylus tip perpendicular to the wear direction. Data acquisition and analysis were performed using Vision64 software V1.0 (Bruker Corporation). For all wear tracks, a cross-sectional profile was obtained in a direction perpendicular to the center of the track, and the track width, depth, and the amount of material accumulation (pile-up) at the edges were determined. The obtained data also enabled the calculation of parameters such as average roughness (Ra), root mean square roughness (Rq), average peak-to-valley height (Rz), total profile height (Rt), maximum peak height (Rp), maximum valley depth (Rv), skewness (Rsk), and kurtosis (Rku).

#### 2.6.2. Scanning Electron Microscopy (SEM)

The morphology of the worn surfaces was examined using a field emission scanning electron microscope (ZEISS GeminiSEM 500, Carl Zeiss AG, Oberkochen, Germany). Prior to imaging, all specimens were coated with palladium–gold (Pd-Au) to ensure the electrical conductivity of the specimen surface. The images were obtained at an accelerating voltage of 5.00 kV and in InLens detector mode at ×50, ×300, and ×2500 magnifications.

#### 2.6.3. Energy-Dispersive X-Ray Spectroscopy

The elemental composition of the wear tracks was analyzed using an energy-dispersive X-ray spectroscopy (EDX) detector integrated with the SEM, using EDAX TEAM eZAF Smart Quant software (Team Basic V4.5). The measurements were performed at an accelerating voltage of 20 kV and a detection (takeoff) angle of 35.3°. For each specimen, the elemental composition was determined using the area scan method, covering the entire worn surface within the image frame.

During the preparation of this study, generative artificial intelligence (AI) tools were used to assist in non-scientific aspects of manuscript preparation. Specifically, Claude Sonnet 5 (Anthropic) was used to assist with the literature search and retrieval support, and ChatGPT (GPT-5.6 Luna, OpenAI) was used to assist in the preparation and formatting of figures. These tools were not used to generate, analyze, or interpret any scientific data, and were not involved in the study design, experimental work, or the scientific conclusions of this study. The author has reviewed and verified all AI-assisted content and takes full responsibility for the accuracy and integrity of the manuscript.

#### 2.6.4. Density and Porosity Measurement

In order to calculate the porosity values after printing of the samples, density measurements were performed both before printing (using pieces taken from different points of the filament) and after printing (on FDM-printed test specimens). The porosity of the printed samples was calculated based on the density of the filament and the relative density reduction in the printed samples (Porosity (%) = (1 − ρ_printed/ρ_filament) × 100).

## 3. Results

### 3.1. Mechanical Properties

Prior to mechanical and tribological characterization, the density of the printed samples was measured by the Archimedes principle to determine the porosity. The density of the filament before printing was measured as an average of 1.334 ± 0.012 g/cm^3^ for samples taken from six different locations, while the average density values obtained from five different test specimens after printing were found to be 1.249 ± 0.010 g/cm^3^. According to these values, the calculated porosity value was 6.37 ± 0.48%. This result is due to the fact that there is typically a limited level of internal porosity in FDM prints, despite using 100% infill. These limitations are consistent with the inherent nature of additive manufacturing.

The tensile and compression stress–strain behaviors of neat PETG and PETG/hBN composites (1, 3, and 5 wt% h-BN) are shown in Figure 7 and Figure 8. The mechanical properties calculated from the numerical data of these curves are summarized in Table 3 and Table 4.

From the tensile test (Figure 7), neat PETG and PETG/1hBN exhibited the highest yield and tensile stresses, reaching values of 65.46 and 65.67 MPa, respectively. This was followed by a strain-softening region. Beyond this point, neat PETG exhibited a stable and long tensile plateau extending up to approximately 89% strain and then underwent ductile deformation. In contrast, with the addition of h-BN, ductility gradually decreased after the yield point. Although the elongation at break of PETG/1hBN was about 42%, those of PETG/3hBN and PETG/5hBN were considerably lower, being 24% and 30% respectively.

In the compression test (Figure 8), the neat PETG and h-BN-filled PETG showed a glassy/semi-crystalline behavior characteristic of thermoplastics, in which yielding is followed by strain hardening and no catastrophic failure occurs up to the range tested. The compressive yield stress for PETG/1hBN was the highest among all of the four compositions, having a value of 48.8 MPa. The yield stress value was the lowest for PETG/3hBN with a value of 42.26 MPa.

### 3.2. Tribological Properties

#### 3.2.1. Coefficient of Friction (COF)

The bar graph of the average coefficient of friction obtained at different applied loads for neat PETG and PETG/h-BN composites is given in Figure 9. At all tested loads, it is evident that PETG/1hBN has a significantly lower average COF value compared to neat PETG and the other composite ratios. At a normal load value of 5 N, the average COF was at a level of 0.49 for neat PETG, while it decreased to 0.26 for the PETG/1hBN composite. However, for PETG/3hBN and PETG/5hBN, these values only decreased to 0.40 and 0.46, respectively. Similarly, under a regular loading value of 10 N, values of 0.51, 0.34, 0.42, and 0.44 were recorded for PETG, PETG/1hBN, PETG/3hBN, and PETG/5hBN, respectively, and in the case of 15 N, values of 0.53, 0.31, 0.47, and 0.53, respectively, were recorded. The maximum reduction value in the COF among all the compositions was observed in the PETG/1hBN composite, giving a reduction of up to 46%. Significantly, PETG/3hBN and PETG/5hBN did not yield a consistent improvement as compared to neat PETG.

Table 5 presents the standard deviations and coefficient of friction values across all compositions and loads. Considering the standard deviations, coefficient of friction, and difference values, the standard deviations indicate that the repeatability is good. The average COF difference between PETG/1hBN and neat PETG exceeds the corresponding standard deviations by a large margin at each load, supporting that the observed reduction is statistically significant. In contrast, the COF values of PETG/3hBN and PETG/5hBN significantly overlap with each other and, at some loads, approach those of neat PETG within their combined uncertainties. This is consistent with the more inconsistent tribological behavior discussed in Section 4.2.

The variation in the coefficient of friction with sliding distance for 5, 10, and 15 N normal loads is shown separately in Figure 10, Figure 11 and Figure 12, respectively. For all applied normal load values, neat PETG reaches a relatively high COF plateau after a short running-in period and remains at approximately the same level. PETG/1hBN, on the other hand, has the lowest steady-state COF value and was determined to be the composition with the longest running-in period. This can be characterized as the formation and gradual development of a stable transfer film layer. PETG/3hBN and PETG/5hBN settled at values between neat PETG and PETG/1hBN and exhibited a scattered and fluctuating coefficient of friction curve. PETG/5hBN, at a normal load of 15 N, approached the coefficient of friction value reached in the neat PETG curve and exceeded the coefficient of friction value of neat PETG at certain points. This indicates that a high h-BN content is intermittently balanced by a less consistent and unstable transfer film layer due to filler material agglomeration. This type of behavior is in line with what Joshi et al. [32] previously reported for PAEK/h-BN nanocomposites; it was noted that in cases in which a specific ratio of fillers is reached, particle agglomeration degrades the transfer film, causing more wear.

#### 3.2.2. Surface Roughness of Wear Tracks (Profilometry)

The two-dimensional profilometer cross-sections obtained from the wear tracks formed on the specimen surfaces as a result of the wear tests conducted under 5, 10, and 15 N loads are shown in Figure 13, Figure 14 and Figure 15. At all loads, the wear track of PETG/1hBN remained significantly shallower and narrower compared to the other three compositions. The pile-up accumulated at the edges of the track was also 4–5 times lower compared to neat PETG. To explain this numerically, the average roughness value Ra for PETG/1hBN in the wear tests conducted at 5, 10, and 15 N loads was 0.130, 0.276, and 0.442 µm, respectively, whereas these values were much higher for neat PETG at the same loads (0.533, 1.728, and 2.014 µm). In contrast, neat PETG, PETG/3hBN, and PETG/5hBN specimens exhibited significant pile-up on both edges of the wear track, particularly in the wear tests conducted at 10 N and 15 N normal loads, and deeper and more irregular wear tracks were observed. In these three specimens, the peak-to-valley height (Rz) and total profile height (Rt) reached levels of 18–21 µm.

Compared with neat PETG, it is noteworthy that PETG/3hBN did not show a better result in terms of roughness and even left a rougher wear track than neat PETG at 5N. This result is consistent with the moderate-to-poor tribological performance observed in the COF measurements. These trends provided consistent results at all three loads and are consistent with the COF and SEM observations. Indeed, among the four compositions, the specimens containing 1% h-BN consistently had the smoothest, shallowest, and most stable wear track. In contrast, a proportional improvement in the wear response could not be achieved at higher filler ratios (3% and 5%). When these findings are examined, they confirm that the high filler ratio increases agglomeration in the polymer, resulting in the formation of an irregular transfer film and consequently an unstable and irregular wear track.

#### 3.2.3. SEM Analysis of Worn Surfaces

In Figure 16, the wear surfaces of neat PETG and h-BN-filled PETG composites under the wear loads of 5, 10, and 15 N are comparatively presented in terms of wear track widths and pile-up regions based on SEM images at ×50 magnification. The general trend shows that the wear track width increases with increasing applied load in all samples, and accordingly, the size and amount of the pile-up regions also increase. This indicates that the increase in contact pressure leads to an increase in plastic deformation and, consequently, an increase in material transfer. This is consistent with the findings reported for thermoplastics such as ABS and PLA produced by FDM, indicating that wear systematically increases with load [18].

The neat PETG sample (Figure 16a–c) exhibited the largest wear track and the most severe pile-up behavior at all loads. Particularly under the 15 N load (Figure 16c), pile-up regions of different sizes formed at the track edges and exhibited a highly irregular pile-up characterization. It is known that in unfilled polymers, due to the inability to form a protective film layer, subsurface deformation and cracking mechanisms lead to the formation of large wear layers on the order of several micrometers [21]. The large and irregular pile-up regions in Figure 16c are consistent with this mechanism.

At all load levels, relatively distinct narrower wear tracks are observed in the PETG/1hBN composite. Notably, no pile-up was encountered at 5 and 10 N loads, and only a very small amount of pile-up was observed at 15 N load. This behavior is consistent with the literature findings indicating that h-BN particles form a thin, coherent, and surface-adhering transfer film layer during sliding, thereby reducing friction and wear [20,32,33,38]. At low h-BN loading, the good anchoring of nanoparticles to the counterface asperities increases the bond strength between the transfer film and the surface, preventing the film from being easily peeled off [20], which explains the pile-up-free/low-pile-up surfaces observed in PETG/1hBN.

In PETG/3hBN composites (Figure 16g–i), the pile-up behavior becomes prominent again, with distinct and visibly noticeable pile-up regions forming at all three different wear loads. At 15 N load, a large pile-up region at the beginning of the track, together with cracking/branching along the track line (Figure 16i), was observed. In the literature, it has been shown that when a certain filler ratio is exceeded, particle agglomeration reduces the interfacial bond strength and the transfer film is more easily peeled from the surface [20,35]. The high pile-up and cracking observed in Figure 16i indicate the effect of agglomeration and weak interfacial bonding at this ratio.

Although the wear tracks for PETG/5hBN appear narrower in width compared to neat PETG at low magnification, they exhibit distinct pile-up regions at all load levels. The wear track depth parameters (Rz, Rt) are reported in Section 3.2.2 and show that the overall wear severity is, in fact, at a comparable level to that of neat PETG rather than being genuinely reduced. This is consistent with the evidence related to the h-BN agglomerate observed on the unworn PETG/hBN surface, as presented in Figure 16. It is supported that the underlying cause of this behavior is agglomeration. In addition, this finding is consistent with the “optimum filler ratio” finding also reported in PAEK/hBN and PA6/GF/hBN systems. While wear resistance increases at low h-BN ratios, generally, when a critical ratio of around 1–2% is exceeded, agglomeration occurs due to the reduced distance between particles, and this formation creates weak points at the matrix–particle interface, reducing the quality of the film and wear resistance [32,35].

As a result, the qualitative observations made in Figure 16 show that the PETG/1hBN composite represents the optimum filler ratio in terms of wear resistance within the scope of this study. This finding is also supported by the transfer film formation and controlled fragmentation at high loads observed in PETG/1hBN at ×300 magnification.

The 300× and 2500× magnification details are provided in Figure 17 in order to resolve the sub-micron scale features in the SEM images given at 50× magnification. Here, the details of the wear surfaces of the four compositions under a constant 10 N load are explained. When the 2500× magnification SEM image of neat PETG in Figure 17 is examined, it shows that the dark subsurface void is surrounded by stretched/torn structures and irregular boundaries.

This appearance shows that when the neat polymer cannot develop a protective transfer film, wear develops through subsurface crack propagation and therefore causes the formation of large and irregular wear sheets. When the 2500× magnification images of the PETG/1hBN composite in the wear region are examined, it is seen that the flake structure has a sharp and regular boundary. This morphology is consistent with the finding that h-BN layers slide over each other to form a regular tribofilm and are transferred as a thin layer [33]. In a study in which h-BN was incorporated at micro- and nanoscale sizes, it was shown that compared to the thick, layered and discontinuous film in the micro composite, the nanocomposite had a thin, coherent and uniform structure, and that this difference effectively reduced friction [38]. The relationship of the regular flake boundary observed for PETG/1hBN in Figure 17, with the low wear track width and almost no pile-up clearly indicated in Figure 16d–f, supports this finding. In the PETG/3hBN and PETG/5hBN images, irregular fractured surfaces and discontinuous crack lines are observed. This is consistent with the finding that when the optimum filler ratio is exceeded, agglomeration weakens the interfacial bond strength and the transfer film is more easily peeled off [20,32]. Similarly, in the PA6/GF/hBN system, it has also been shown that fiber–matrix separation increases at high h-BN ratios [35].

An additional SEM analysis was performed on the unworn surface to further examine the distribution of the filler material prior to the wear test. As shown in Figure 18, a distinct and relatively large agglomerated h-BN residue was observed on the surface. This image provides direct evidence of h-BN agglomeration in the composite.

#### 3.2.4. EDX Analysis of Worn Surfaces

The elemental analysis was performed by EDX using an area scan across the wear track width of each specimen, covering the wear surface. The EDX results of the wear surface for each composite are given in Table 6. In the EDX results, only C and O elements were detected for neat PETG, whereas B and N, which are the characteristic elements of h-BN, were detected in all h-BN-containing composites. This indicates that the h-BN particles did not leave the surface after wear and remained within the sliding region [38]. Similarly, in the literature, EDS analysis has been performed on the wear surfaces of h-BN-containing composites, and the presence of h-BN was confirmed by the increase in the N signal. In addition, Panda et al. [38] detected B and N signals in their XPS analyses performed on the tribofilm and demonstrated that h-BN was present within the tribofilm after wear.

The increase in the amounts of B and N in the wear region with increasing h-BN content should not, by itself, be considered an indicator of tribofilm formation or an improvement in wear resistance. Due to the low atomic numbers of B and N, their detection in EDX analyses involves low-energy X-rays, and factors such as absorption and detector efficiency may therefore contribute to uncertainty in the results. Indeed, Vikram et al. [35] confirmed that the B atom could not be detected by the detector in their EDX analyses and that fluctuations could also occur in the data for the N atom. For this reason, EDX measurements were performed at three different points for each wear condition, and the results are presented as mean ± standard deviation. Therefore, in the present study, the EDX results were primarily evaluated as elemental evidence demonstrating the presence of h-BN on the worn surface; they were not considered as a standalone quantitative indicator of tribofilm formation or wear resistance. Instead, the EDX results were considered together with the SEM, profilometry, and COF results when interpreting the differences in tribological performance among the compositions.

#### 3.2.5. EDX Analysis of Worn Surfaces

In order to verify the adequacy of the homogeneous distribution on the printed sample for compositions with all h-BN ratios, SEM imaging and EDX elemental (B K/N K) mapping were performed on the unworn (as-printed) surfaces of PETG/1hBN, PETG/3hBN, and PETG/5hBN samples (Figure 19). The B/N-associated signal covered approximately 9%, 10%, and 8% of the mapped area, respectively. In the PETG/1hBN ratio, a micrometer-scale agglomerate was observed within a background consisting of sparsely and finely distributed B and N (Figure 19a,b). This is consistent with the isolated agglomerate also detected in PETG/5hBN. In contrast, PETG/3hBN and PETG/5hBN exhibited a denser, finer, and more homogeneously distributed B/N signal across the mapped region (Figure 19c–f). No distinct cluster of similar size was observed within the same imaging field. Considering the limited imaging area and single-region sampling inherent to this analysis, these observations should be interpreted as qualitative evidence of the presence and local distribution state of the filler rather than as a statistically representative, quantitative dispersion metric.

Notably, the B/N-associated area coverage did not increase monotonically with h-BN content (9%, 10%, and 8% for 1, 3, and 5 wt%, respectively). EDX elemental mapping cannot provide a continuous concentration scale and categorically classifies each pixel according to the detection threshold. Therefore, this area fraction reflects the spatial extent of filler-containing regions rather than the total amount of filler. The concentration of h-BN particles into increasingly fewer and denser agglomerates rather than being spread over a proportionally larger area at higher nominal ratios supports the agglomeration-based interpretation given in Section 4.2. Therefore, despite the higher mass fraction of filler, the total mapped area fraction does not vary directly in proportion to the nominal filler content.

Representative EDX spectra for PETG/1hBN, PETG/3hBN, and PETG/5hBN, showing the characteristic B and N peaks used to generate the quantitative results in Table 6, are provided in Figure 20.

## 4. Discussion

### 4.1. Mechanical Properties

The results obtained from the tensile and compression tests indicate that the low filler content is the optimum and most balanced composition in terms of mechanical performance in PETG/h-BN composites produced by FDM. Compared with neat PETG, PETG/1hBN exhibited almost unchanged UTS (65.67 MPa) and elastic modulus (1021.1 MPa). However, among the four parameters, it reached the highest compressive yield strength (48.8 MPa). This behavior suggests that the addition of 1 wt% h-BN resulted in nearly unchanged tensile strength and elastic modulus compared with neat PETG, while the improvement observed in compressive yield strength indicates a more pronounced mechanical effect under compression. This observation is consistent with the results reported by Chen et al. [39] for epoxy/h-BN nanocomposites. In their study, it was shown that the addition of only 1 wt% h-BN increased the UTS by 6.6% and the elastic modulus by 5.5% compared to neat epoxy. This improvement was attributed to the high elastic modulus of the nanofiller (h-BN), which ranges between 400 and 900 GPa depending on its crystalline structure.

A porosity of 6% was measured as a result of the density measurements. Despite using 100% infill during printing, this indicates that the FDM-processed parts contain a level of internal voidage that is typical for FDM-printed parts. This porosity manifested itself as lower mechanical properties after FDM printing compared to plastic injection molding.

However, the effects of h-BN addition on elongation at break are quite contradictory in the literature. The ductility loss tendency observed in our study (a decrease from 89% to the range of 24–30% with increasing h-BN content) is in agreement with the results reported by Mistry and Randhawa [34] for PA66/h-BN composites. In a more direct comparison with another study, Ralls et al. [46] demonstrated that the addition of Al_2_O_3_ and h-BN to an ABS matrix by interlayer in situ spraying resulted in decreases in UTS (17.17% reduction), ductility, and toughness. This decrease was explained by the weak interfacial adhesion between the h-BN particles and the polymer, as well as micro-porosity formed during diffusion. Due to its production by FDM and its inclusion of h-BN filler, this study shows considerable methodological similarity to the present research. With increasing h-BN content, a significant decrease in elongation at break was observed. This can be associated with the rigid and relatively brittle structure of the h-BN particles. These brittle particles limit the deformation of the PETG matrix and accelerate crack initiation and deformation by creating stress concentration regions under tensile loading. At higher filler contents, stress concentration increases due to the agglomeration of the particles, resulting in a significant decrease in ductility.

On the other hand, Randhawa and Patel [36] reported opposite trends, with increasing ductility, in the nylon 6/h-BN system, while Vikram et al. [35] reported similar trends in the PA6/glass fiber/h-BN system. These differences can be explained by system-specific factors, such as the already brittle effect introduced by glass fiber reinforcement and the moisture sensitivity of nylon. Overall, this comparison demonstrates that the net effect of h-BN incorporation into the polymer matrix on mechanical behavior is strongly dependent on system-specific factors, such as the chemistry of the matrix, the manufacturing method, and the quality of interfacial adhesion.

### 4.2. Tribological Performance and Wear Mechanisms

The combined COF, profilometer, and SEM results consistently demonstrate that 1 wt% h-BN provided the most balanced tribological performance among the investigated compositions for FDM-produced PETG composites under ball-on-flat wear conditions. At the investigated filler content providing the most balanced performance, compared to neat PETG, a 40–46% lower coefficient of friction at all loads (Figure 9, Figure 10, Figure 11 and Figure 12), a 4–5-fold reduction in wear track roughness (Figure 13, Figure 16 and Figure 17), and SEM examinations revealed that the pronounced delamination and tearing observed in neat PETG were largely absent, and that a controlled and balanced tribofilm formation occurred. This behavior is consistent with the transfer film mechanism reported in studies conducted on polymers containing solid lubricant additives [19,20,22]. At low reinforcement ratios, h-BN, which has a layered structure, is sufficient to enable the formation of a thin, consistent, balanced, and well-adhered tribofilm that separates the sliding surfaces from each other during wear, reducing abrasive and adhesive wear. However, the filler itself should not be added at sufficiently high ratios to act as a third abrasive. The friction coefficient can be attributed to h-BN’s layered structure along with its low shear strength. When sliding happens, the particles facilitate interfacial shear by creating a lubricating transfer layer between the surfaces. This cuts back on direct polymer-to-counterface contact and lowers friction as a result. The Author did not determine any specific wear rate from weight loss measurements here. The initial tests showed no clear change in specimen mass on the balance, which is reasonable if the loads and sliding distance were not enough to remove much compared to the total mass of the specimen. Some material might accumulate along the edges of the track instead of fully detaching, and other loose particles may be lost during or after the test. That makes mass loss an unreliable measure overall. Wear was evaluated instead through profilometry on the tracks using depth along with the roughness values from the table and figures. It provided a more direct measure of what occurred under the investigated conditions. The resolution of the balance was insufficient for this setup, and it seems like that limited how much they could say about the volume of material removed.

The SEM observations support this mechanism, since the PETG with one h-BN did not have as much delamination or tearing compared to neat PETG. This suggests a more stable sliding interface, consistent with the gradual development of the transfer film discussed in Section 3.2.1.

In composites containing 3% and 5% h-BN, a proportional tribological improvement compared to 1% could not be achieved. This non-monotonic optimum loading behavior has been widely reported in polymer/h-BN composites produced by conventional methods: a study by Joshi et al. [32] demonstrated that reinforcement levels above only 0.5 wt% h-BN in PAEK polymer increased wear by reducing the transfer film effectiveness due to particle agglomeration; similar optimum filler contents have also been reported for h-BN-filled polyimide [33], PA66 [34], PA6/glass fiber hybrids [35], and neat PA6 [36]. The results, obtained for the first time in the present study, for FDM-produced PETG composites demonstrate that they exhibit the same agglomeration-induced optimum loading behavior, even though they are processed by polymer additive manufacturing. This observation is further supported by the SEM image of the unworn PETG/5hBN composite (Figure 18), where a relatively large agglomerated h-BN region can be clearly observed.

When the elemental mapping of the unworn printed sample surfaces (Figure 19) and the agglomerate detected in PETG/5hBN (Figure 18) are evaluated together, they provide direct and qualitative evidence that agglomeration is present within the printed compositions, complementing the previously discussed interpretation based on the property trend of agglomeration.

### 4.3. Mechanical–Tribological Relationship

There is a strong consistency between the decrease in tensile ductility observed with increasing h-BN content and the optimum loading behavior in tribological performance. In composites containing a low amount of h-BN filler (1%), a well-dispersed filler provides both actual mechanical reinforcement (high compressive flow stress) and effective tribofilm formation. However, at higher filler contents such as 3% and 5%, large h-BN agglomerations act both as stress concentrator/crack initiation regions during tensile loading and form an irregular, unstable transfer film in sliding contact. This dual observation strongly supports the conclusion that PETG/1hBN is the composition offering the most balanced performance in both mechanical and tribological aspects among the four compositions tested in this study.

### 4.4. EDX Findings

Elemental analysis performed on the wear surfaces confirmed the presence of boron and nitrogen in all h-BN-filled composite samples and demonstrated that h-BN remained present in the contact region after wear and was not completely eliminated (Table 6). The general trend of the B and N element percentages increased with the nominal filler content, reaching the highest values in the 5% h-BN composites ranged from 3.22 ± 0.20 to 4.19 ± 0.24 wt% while the N content ranged from 2.75 ± 0.24 to 4.18 ± 0.64 wt%. In the neat PETG samples, as expected, only carbon and oxygen were detected, which is consistent with the basic chemical composition of the PETG matrix and confirms that there was no contamination from the counter surface.

The tribological surfaces of the specimens were polished to minimize the effects of FDM-induced surface irregularities. Therefore, the obtained results reflect the intrinsic behavior of the PETG/h-BN composites. However, since the internal structure was produced by additive manufacturing, the regions beneath the surface retain the characteristics of the printed samples.

### 4.5. Preliminary Cost Considerations

The aim was to provide a preliminary basis for the economic feasibility claim made in this study. For this purpose, a simplified comparison was performed regarding the cost of a PETG/1hBN component and an equivalent-volume bronze component. Bronze is a material commonly used in similar lightly loaded sliding-contact applications (e.g., bushings and bearing elements) and was therefore considered as a reference for the comparison. Based on the density difference between the two materials (1.29 g/cm^3^ for PETG/1hBN and 8.80 g/cm^3^ for bronze), approximately 6.82 kg of bronze is required instead of 1 kg of PETG/1hBN to produce a component of equivalent volume. Table 7 summarizes the estimated material and processing costs (excluding VAT) for both production methods. Under the determined cost assumptions, it was determined that the estimated cost of the component produced from PETG/1hBN composite was approximately 86% lower than that of the equivalent-volume bronze component. This comparison is only a preliminary assessment based on current local material and processing costs in Türkiye, rather than a comprehensive economic analysis. It is presented as an order-of-magnitude estimate. Factors such as production volume, mold/equipment amortization, or application-specific performance requirements are not taken into account.

## 5. Conclusions

This study systematically investigated the mechanical and tribological characterization of specimens produced by fused deposition modeling (FDM) from filaments manufactured by adding 1%, 3%, and 5% h-BN by weight into a PETG matrix. Tensile and compression tests, as well as ball-on-flat reciprocating wear tests under normal loads of 5, 10, and 15 N, were performed. As a result, the wear surfaces were evaluated together with contact profilometry, scanning electron microscopy (SEM), and energy-dispersive X-ray spectroscopy (EDX) analyses, and the following results were obtained.

1. In the wear test, the PETG/1hBN composite provided the lowest coefficient of friction (COF) at all test loads (5, 10, and 15 N), and an improvement of approximately 40–46% was observed compared with neat PETG.
2. As confirmed by profilometry measurements performed on the wear surfaces of the wear specimens, it also left a 4–5 times shallower and smoother wear track compared with the other three compositions.
3. The SEM examinations also supported the quantitative findings, and while distinct delamination and tearing regions were observed on neat PETG surfaces at high loads, a thin tribofilm that developed with the load and maintained its presence in a controlled manner under friction was observed on the wear surface of the PETG/1hBN composites.
4. In the tensile test, PETG/1hBN exhibited nearly unchanged UTS and elastic modulus values compared with neat PETG, while in the compression tests, it reached the highest compressive yield stress among the four compositions. This indicates that at low filler contents, the h-BN particles were well dispersed within the matrix and provided a genuine reinforcement effect. Indeed, considering that the wear surfaces are subjected to compressive forces, the increase in compressive stress constitutes a strong advantage.
5. With increasing h-BN content, a significant decrease in tensile ductility was observed due to agglomerated regions acting as stress concentrators.
6. The composites containing 3% and 5% h-BN by weight did not provide a proportional improvement in either tribological or mechanical properties compared with the composites containing 1% h-BN by weight. In fact, under some conditions, they exhibited performance equivalent to or weaker than that of neat PETG. This behavior can be interpreted as an indication that increasing the filler content creates agglomerated regions that have a disadvantageous effect within the matrix.
7. EDX analyses confirmed the presence of boron and nitrogen on the wear surfaces of all h-BN-filled specimens. The contents of these elements were observed to increase with the nominal filler content. This confirms that h-BN permanently retained its presence in the wear contact region and was not completely removed.
8. This study focused on the mechanical and tribological properties of PETG/h-BN composite filaments; since the thermal stability and rheological behavior of the system were not investigated, they are recommended as subjects for future studies.

In conclusion, this study demonstrates that the addition of 1% h-BN by weight to PETG can improve the tribological performance of FDM-printed specimens under the tested dry-sliding conditions without compromising the mechanical integrity of the matrix. When the shallow wear track achieved through the low coefficient of friction and the balanced mechanical properties are evaluated together, the PETG/1hBN composite demonstrates promising tribological performance within the load range (5–15 N) and sliding distance (250 m) tested in this study. Component-level validation under representative service conditions is required before its use in practical applications. As a result, it indicates potential application areas for use in lightly loaded, short-to-moderate-duration sliding-contact components such as gears, bushings, and circular bearing elements produced by the FDM manufacturing method. This scope-limited, screening-level approach to assessing application potential is consistent with comparable studies on self-lubricating FDM-printed composites [46]. The fact that this composite material does not require any additional processing or special equipment in existing production lines for FDM filament production, apart from compound preparation in a twin-screw extruder, makes it a practical and economical candidate for the commercial-scale production of self-lubricating engineering components. However, the disadvantages arising from the tendency toward agglomeration at higher filler contents indicate that similar or higher levels of performance could be achieved by incorporating surface modification or alternative mixing strategies into the system to improve the dispersion of h-BN within the matrix. In addition, by determining the optimum ratio, it is anticipated that compositions with much higher performance can be obtained by investigating synergistic effects between h-BN and different solid lubricants within a hybrid composite approach.

All these considered, the current results should be evaluated within the experimental conditions examined in this study. The tribological tests were performed at normal loads of 5, 10, and 15 N and at a sliding distance of 250 m based on previous studies. Therefore, they do not demonstrate long-term wear resistance or component lifetime performance. A more comprehensive evaluation of the practical durability of PETG/h-BN composites under service conditions requires longer tests, a wider load range, and component-level validation.

## Figures and Tables

**Figure 1:**
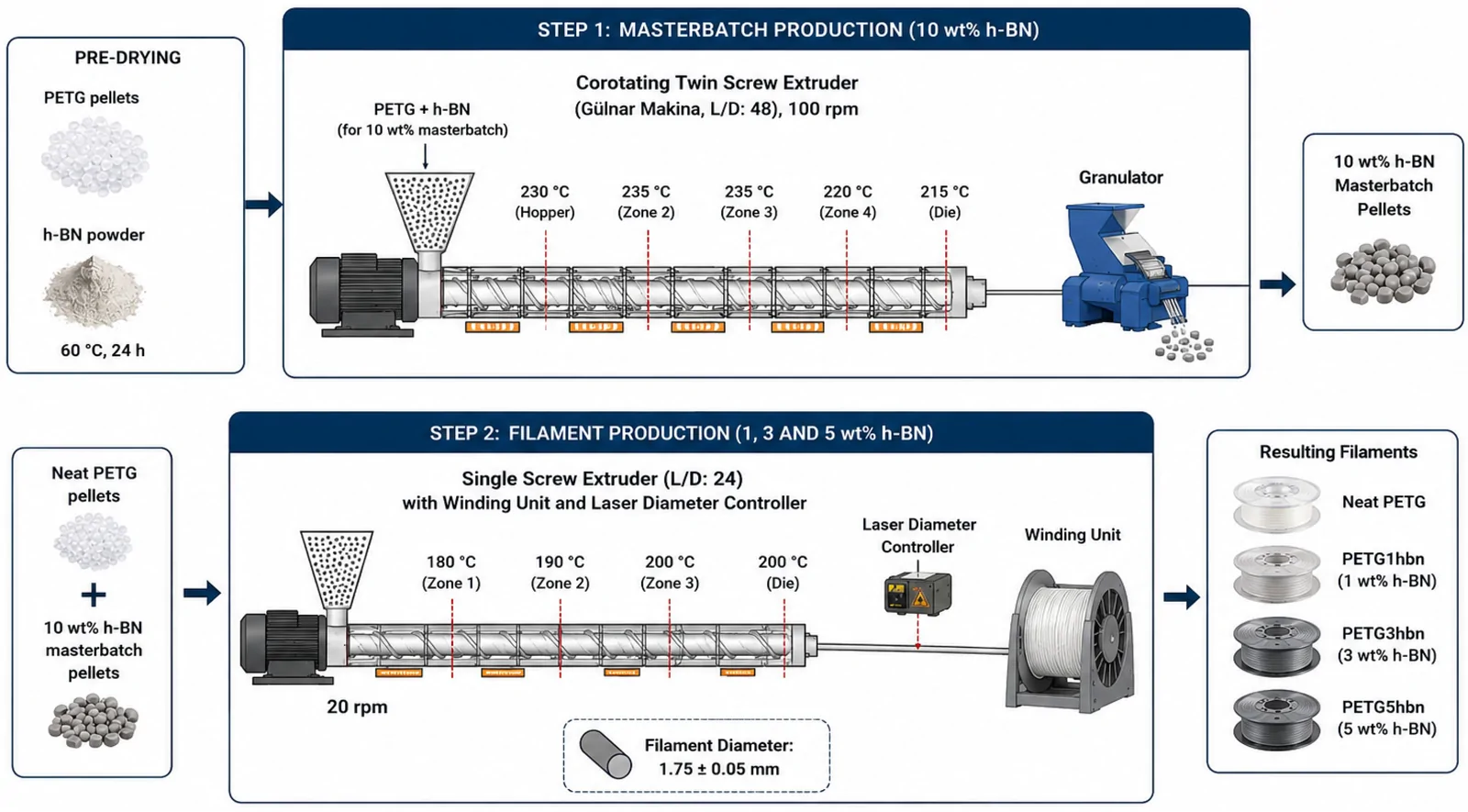
Schematic representation of the two-stage extrusion process for the production of PETG/h-BN composite filaments.

**Figure 2:**
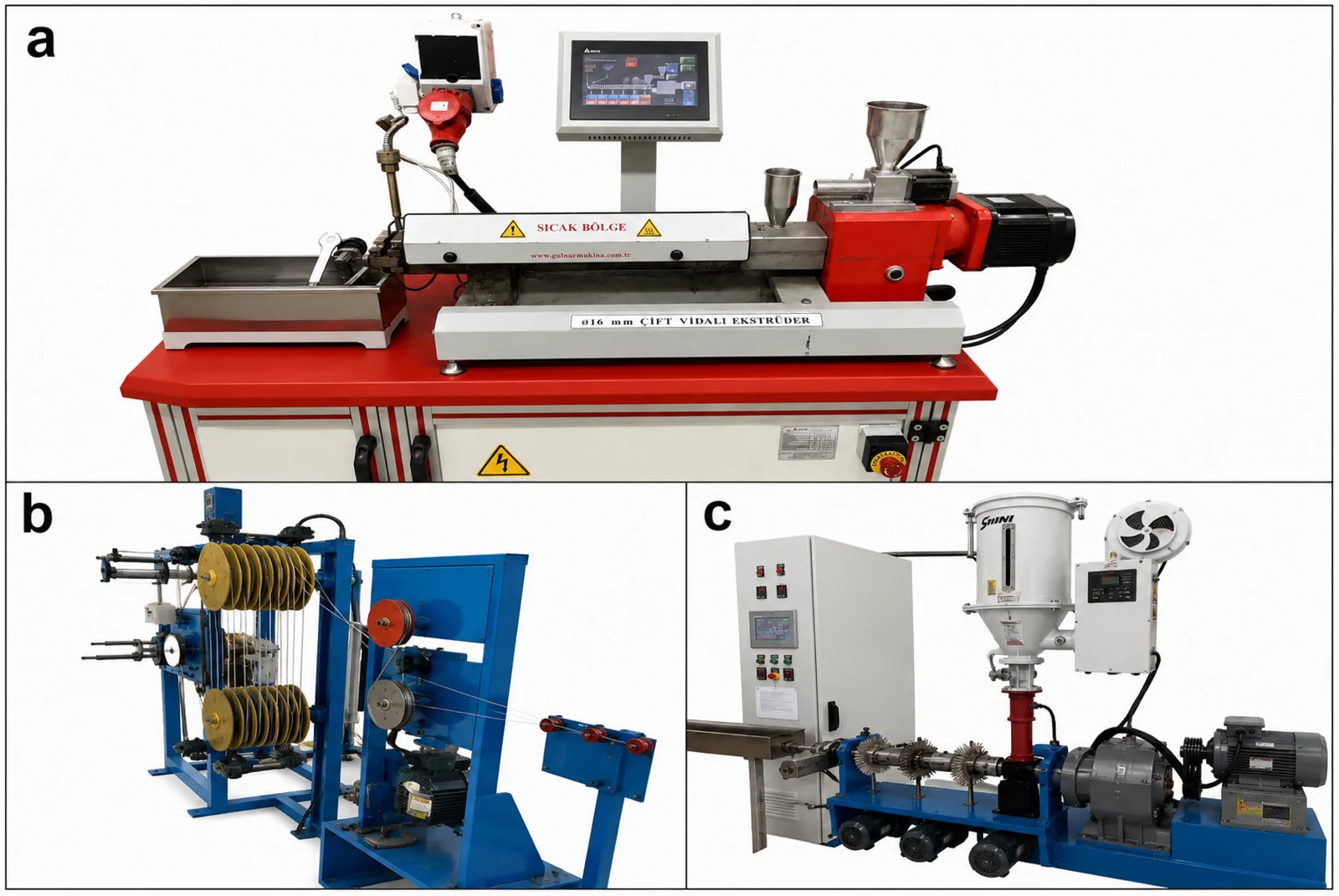
Equipment used for the production of PETG/h-BN composite filaments: (**a**) twin-screw extruder, (**b**) winder and dancer unit of the single-screw extrusion line, and (**c**) single-screw extruder.

**Figure 3:**
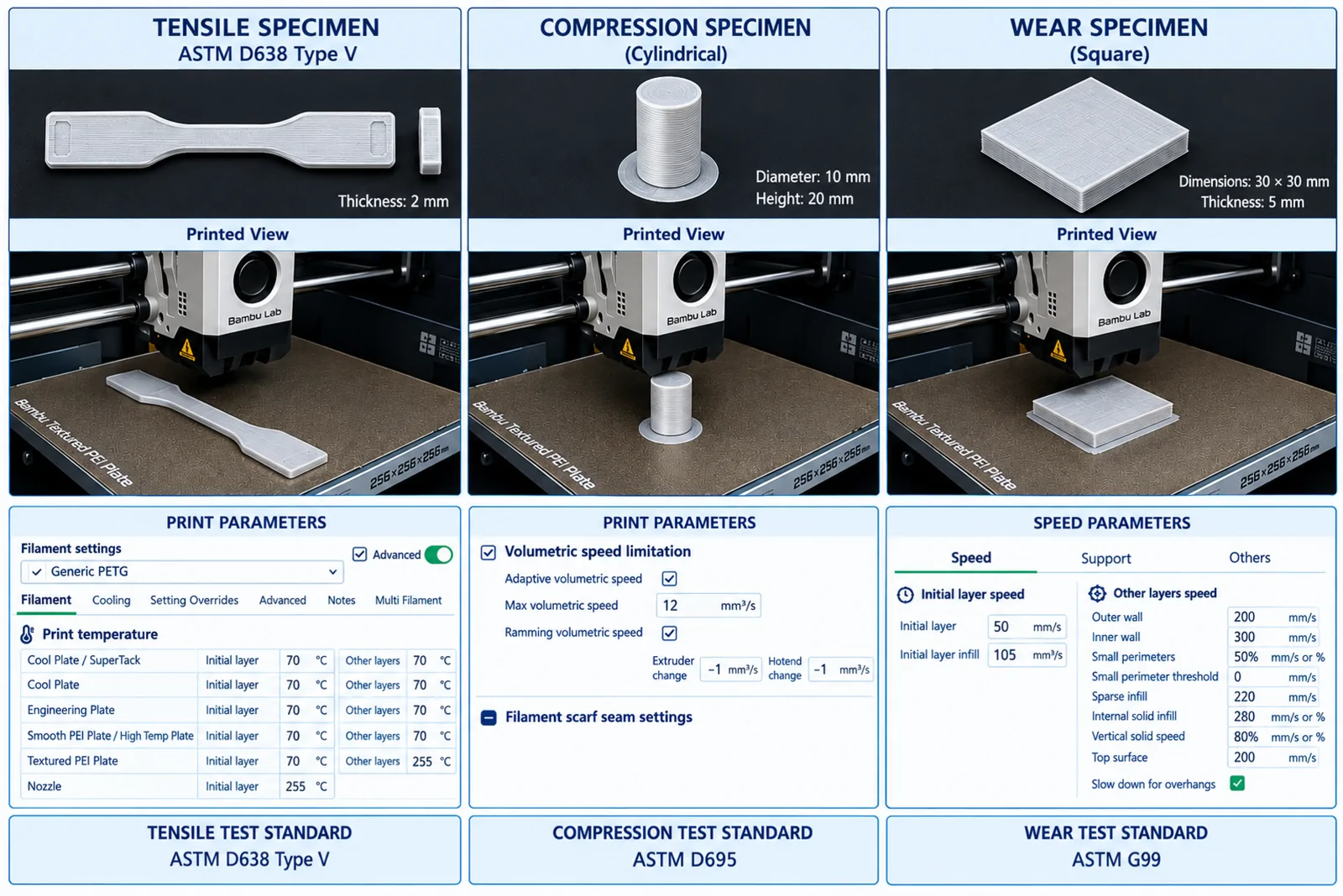
Production method and printing parameter configuration of tensile, compression, and wear specimens produced by FDM.

**Figure 4:**
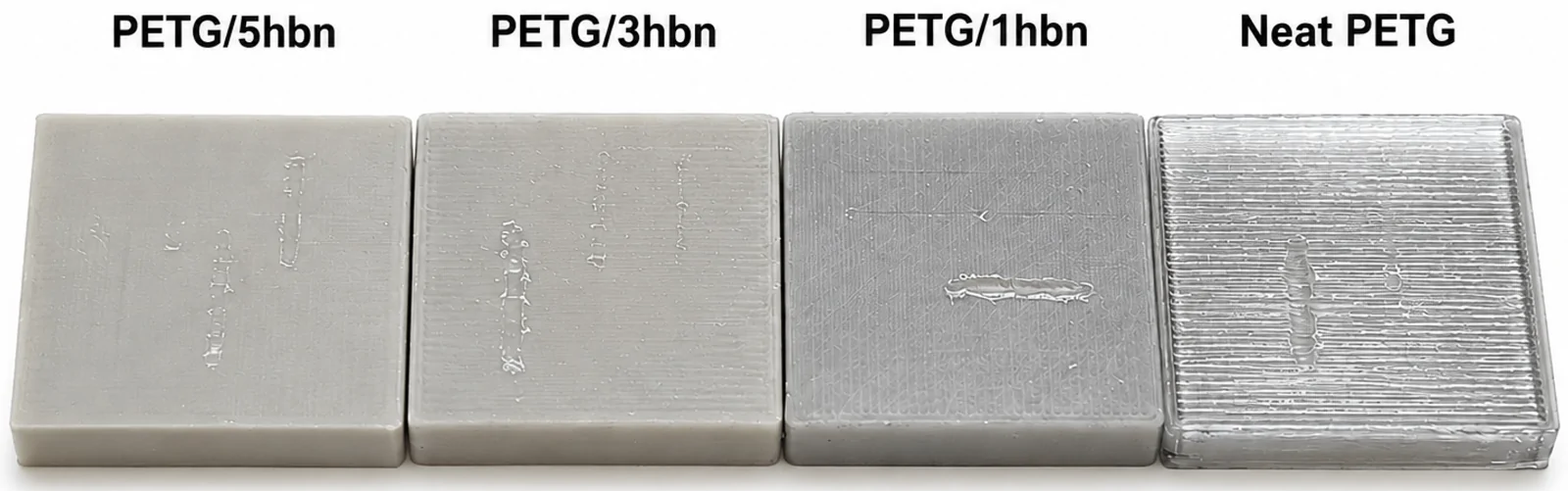
Produced ball-on-flat wear specimens.

**Figure 5:**
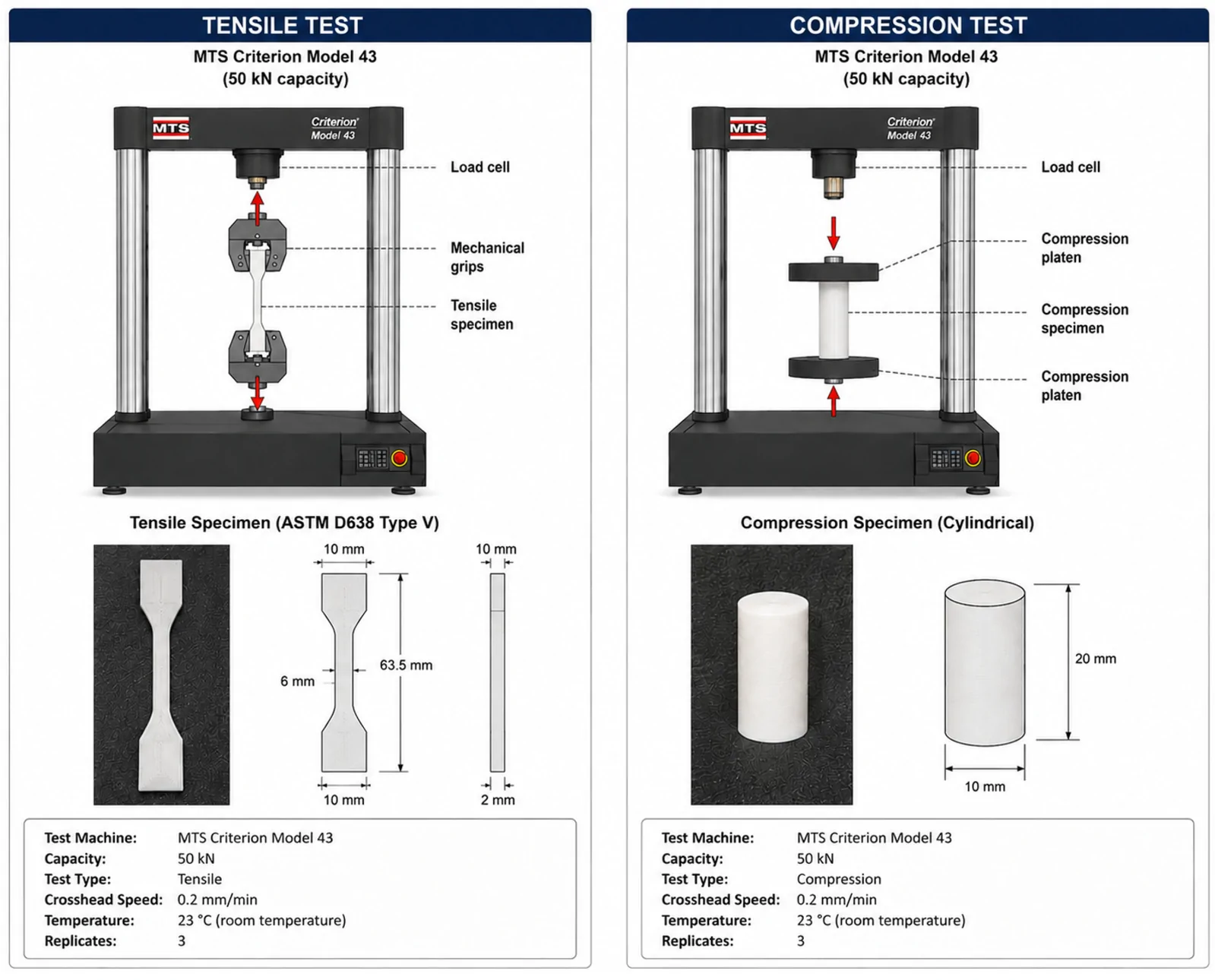
Schematic representation of the tensile and compression test setups.

**Figure 6:**
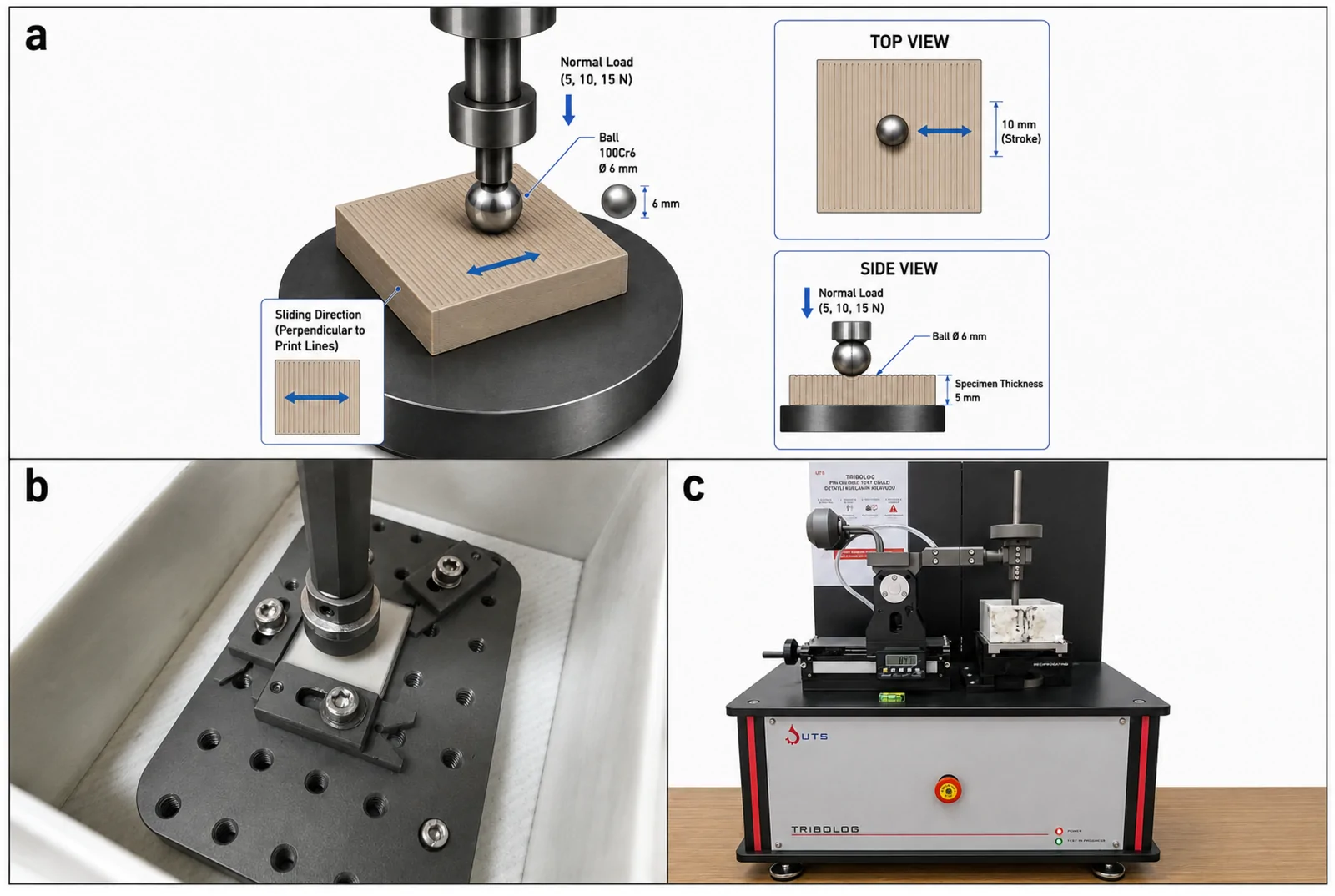
(**a**) Schematic representation of the ball-on-flat wear test configuration, (**b**) worn specimen mounted on the test fixture, and (**c**) UTS Tribolog reciprocating tribometer.

**Figure 7:**
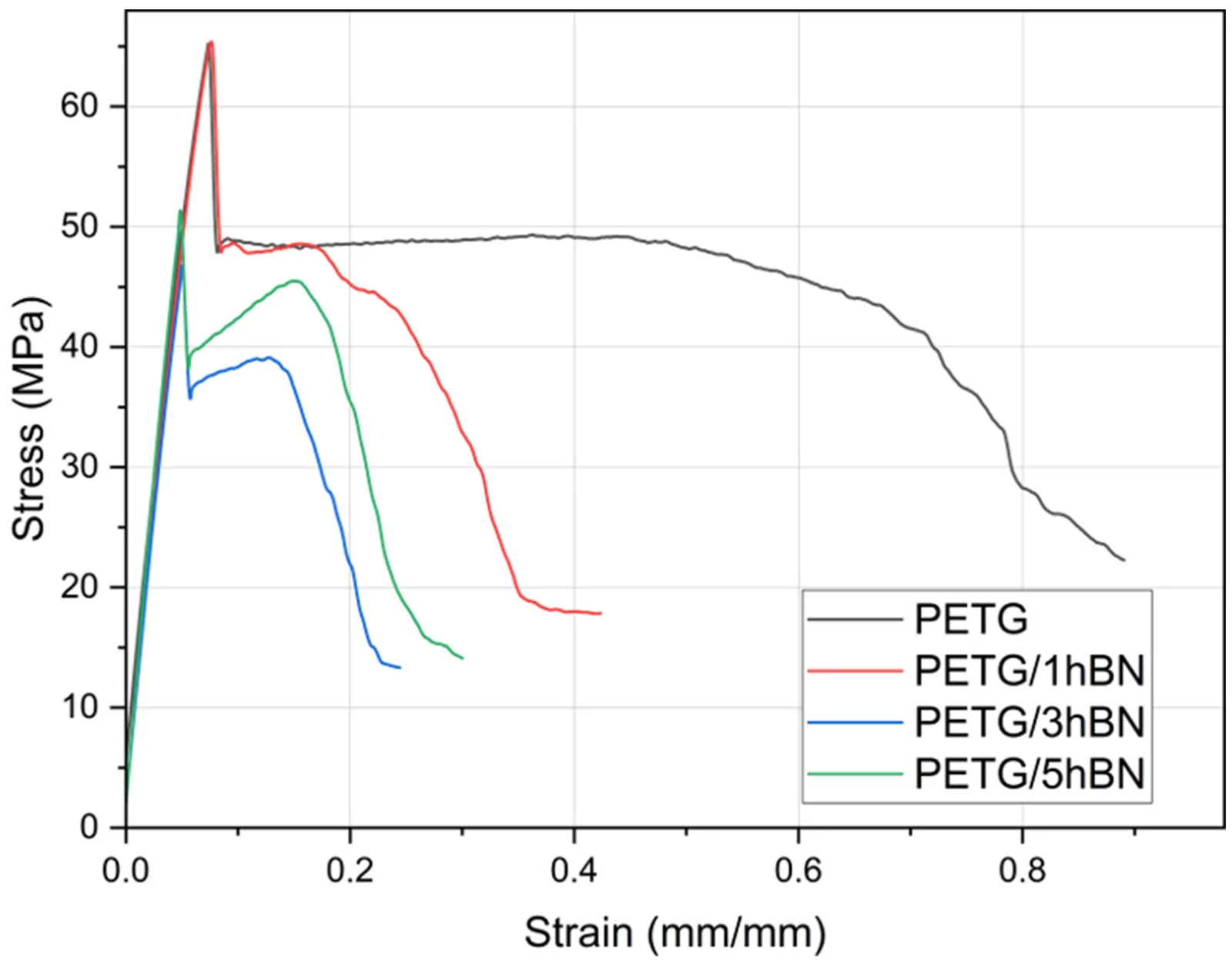
Tensile stress–strain curves of neat PETG and PETG/h-BN composites.

**Figure 8:**
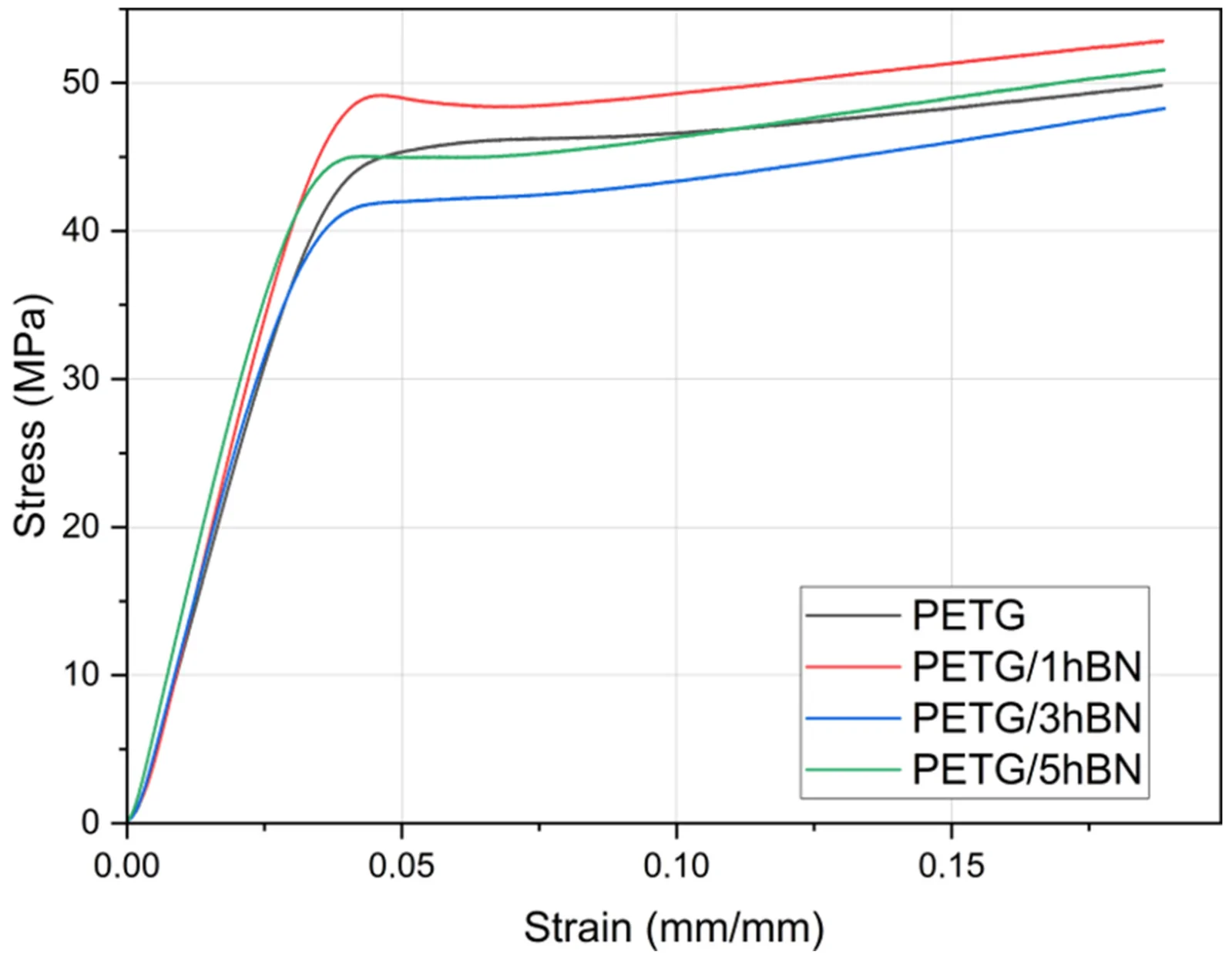
Compression stress–strain curves of neat PETG and PETG/h-BN composites.

**Figure 9:**
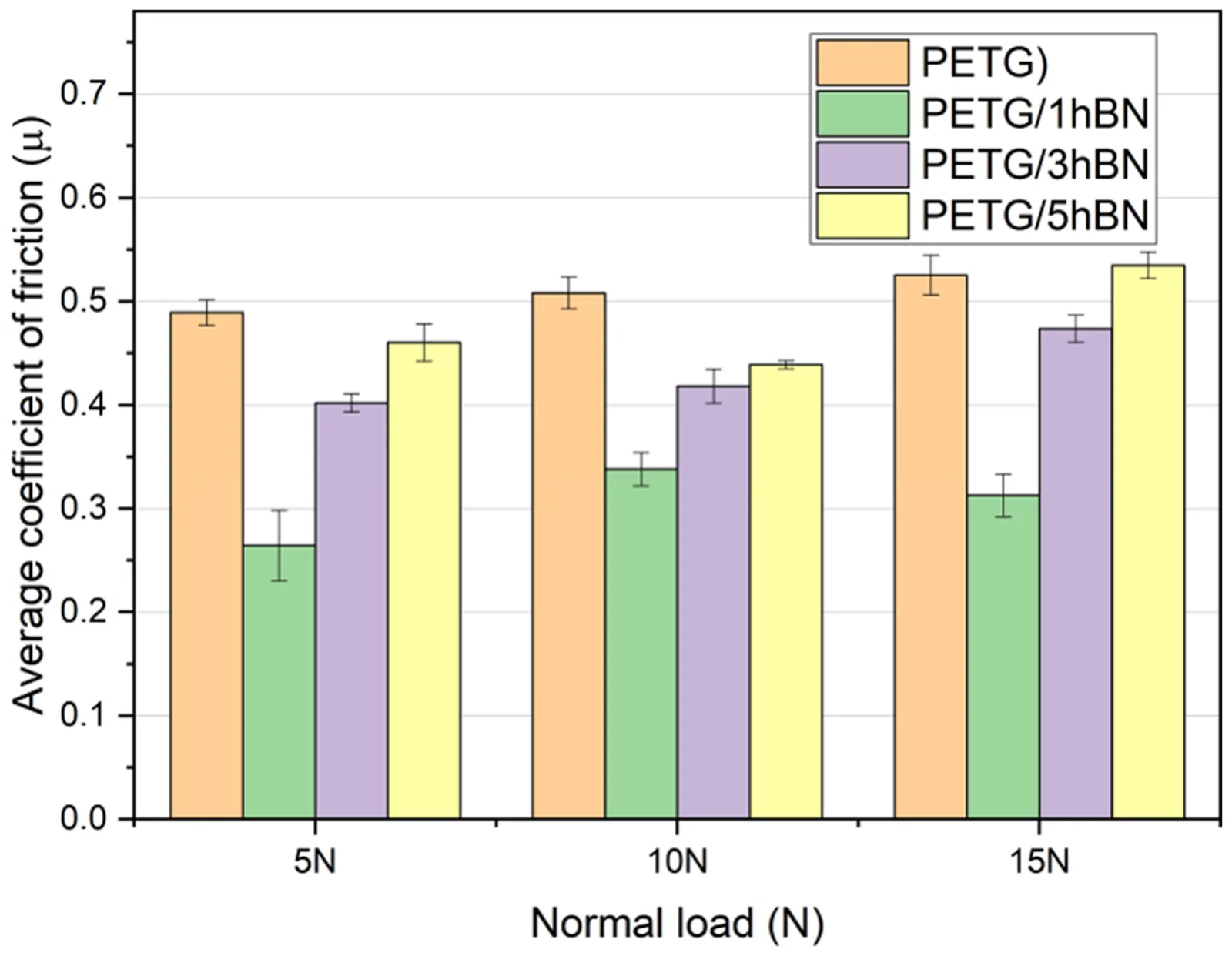
Average coefficient of friction (COF) of neat PETG and PETG/h-BN composites as a function of normal load (5, 10, and 15 N).

**Figure 10:**
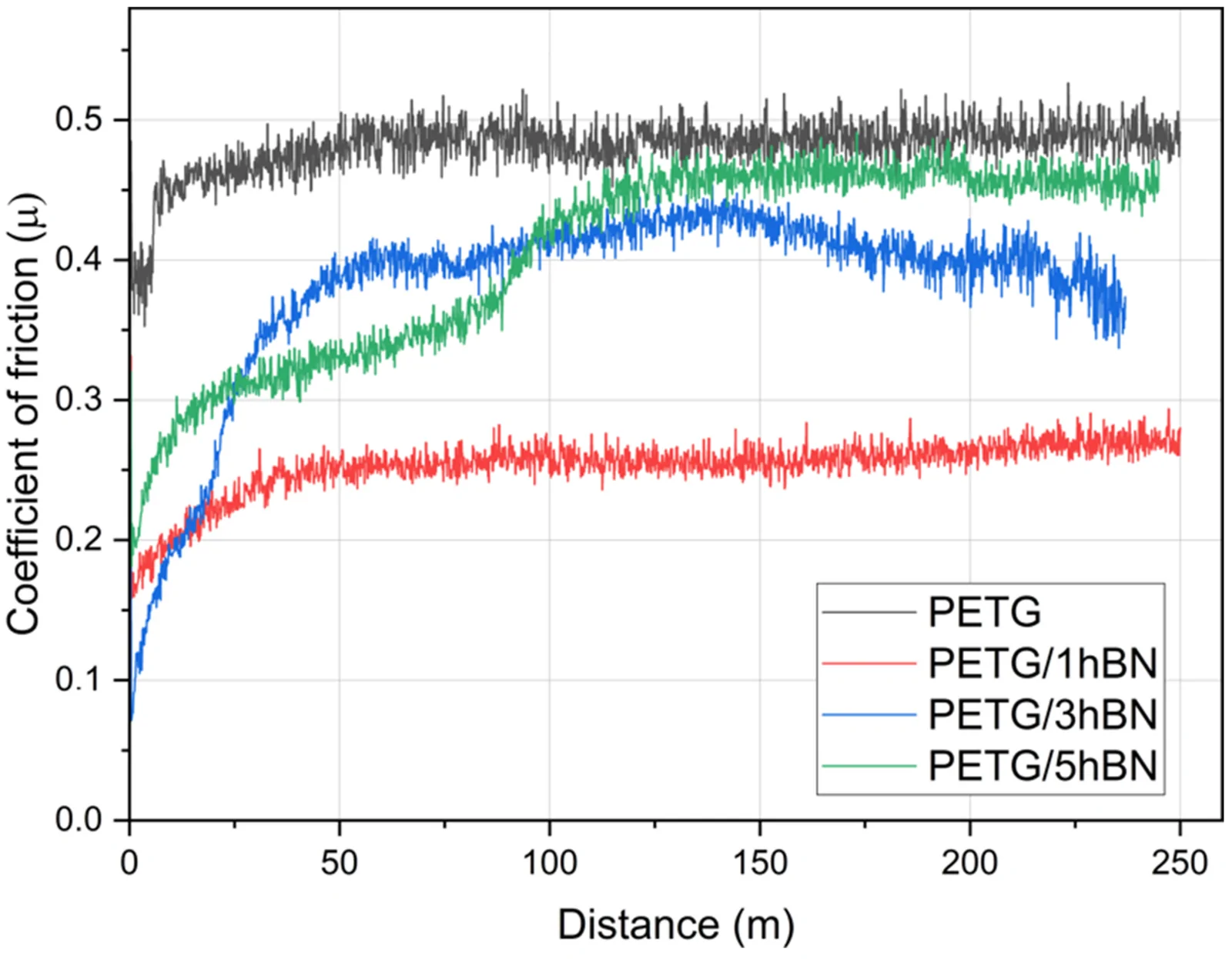
Coefficient of friction as a function of sliding distance for neat PETG and PETG/h-BN composites at 5 N normal load.

**Figure 11:**
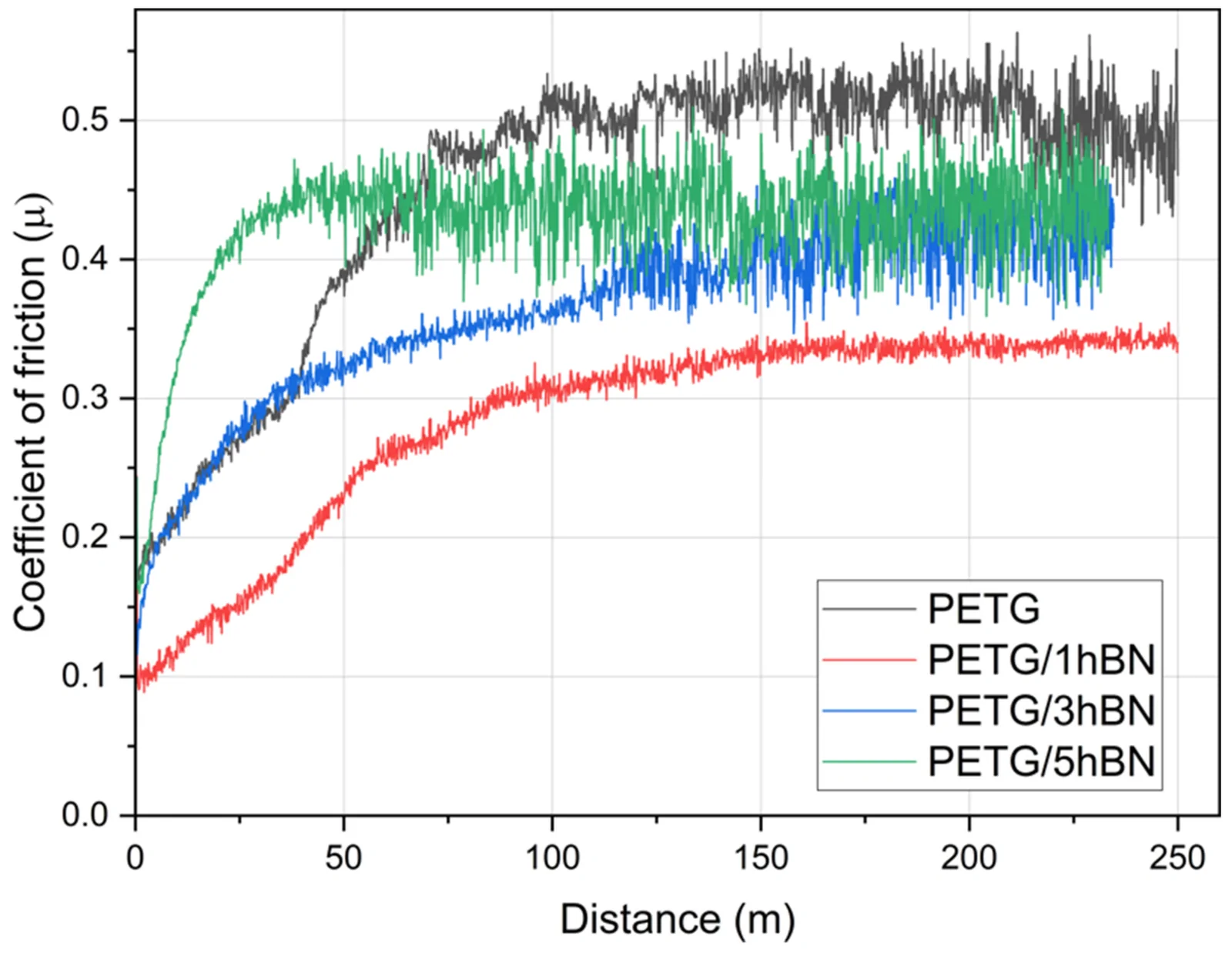
Coefficient of friction as a function of sliding distance for neat PETG and PETG/h-BN composites at 10 N normal load.

**Figure 12:**
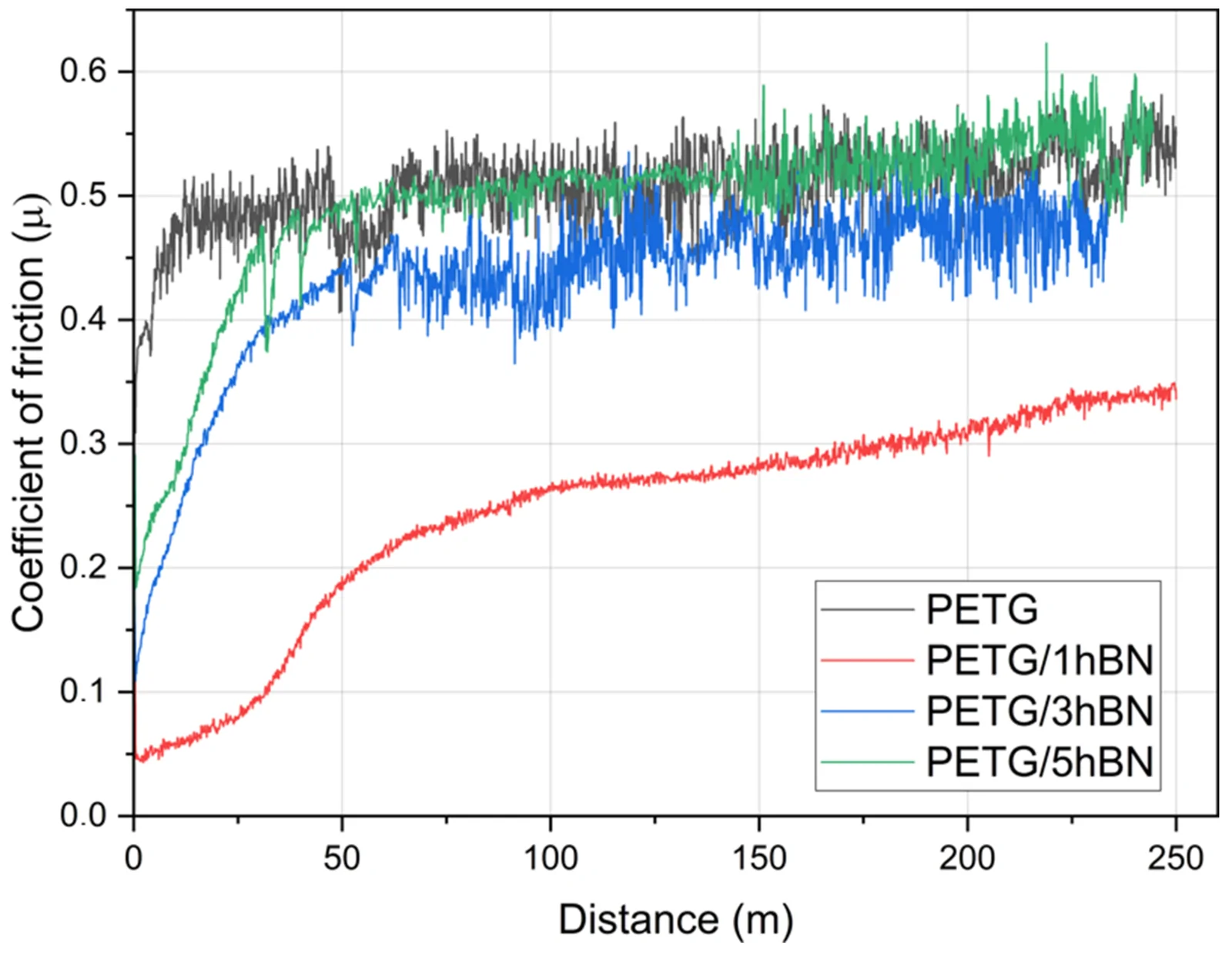
Coefficient of friction as a function of sliding distance for neat PETG and PETG/h-BN composites at 15 N normal load.

**Figure 13:**
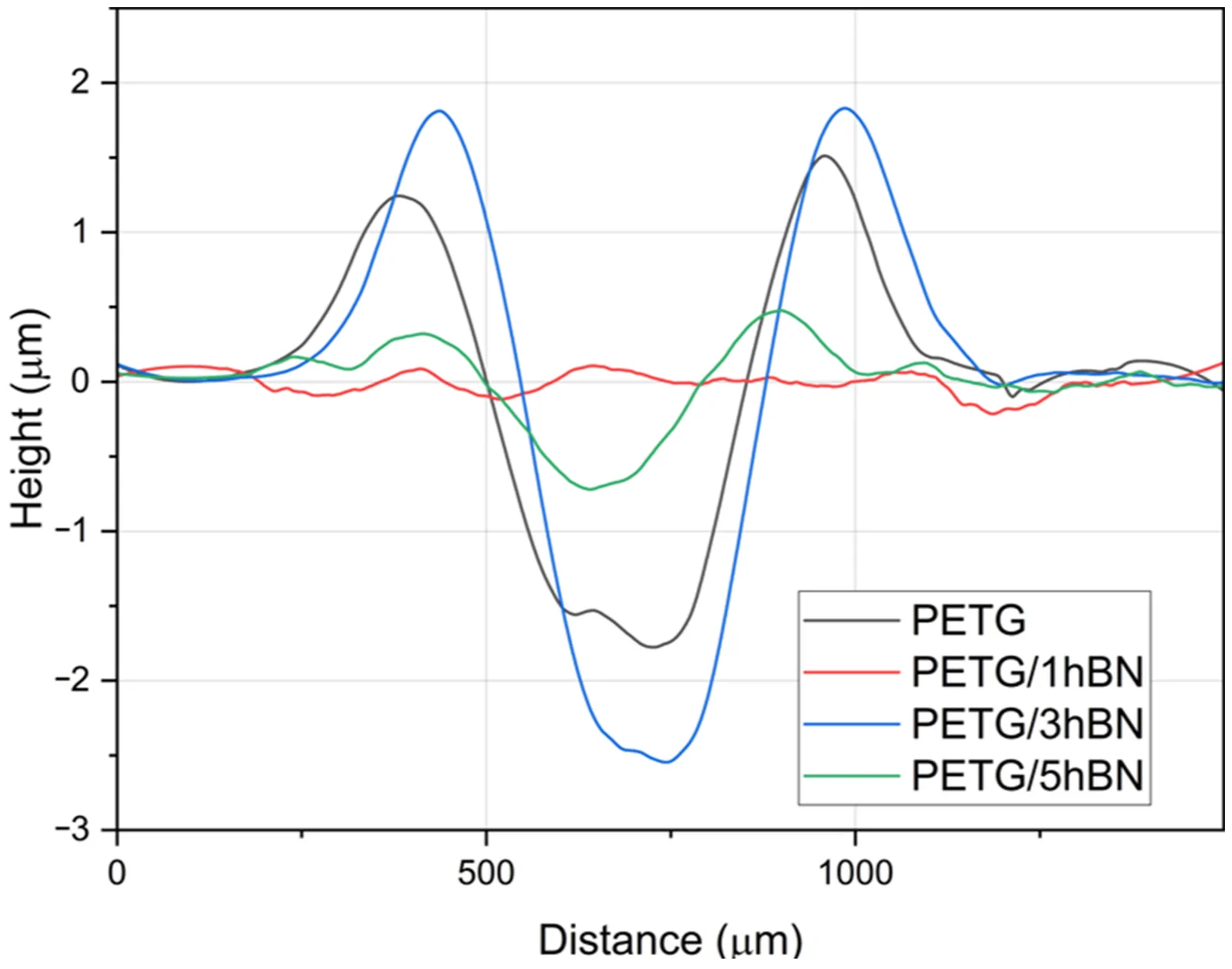
Two-dimensional profilometry cross-sections of the wear tracks of neat PETG and PETG/h-BN composites at 5 N normal load.

**Figure 14:**
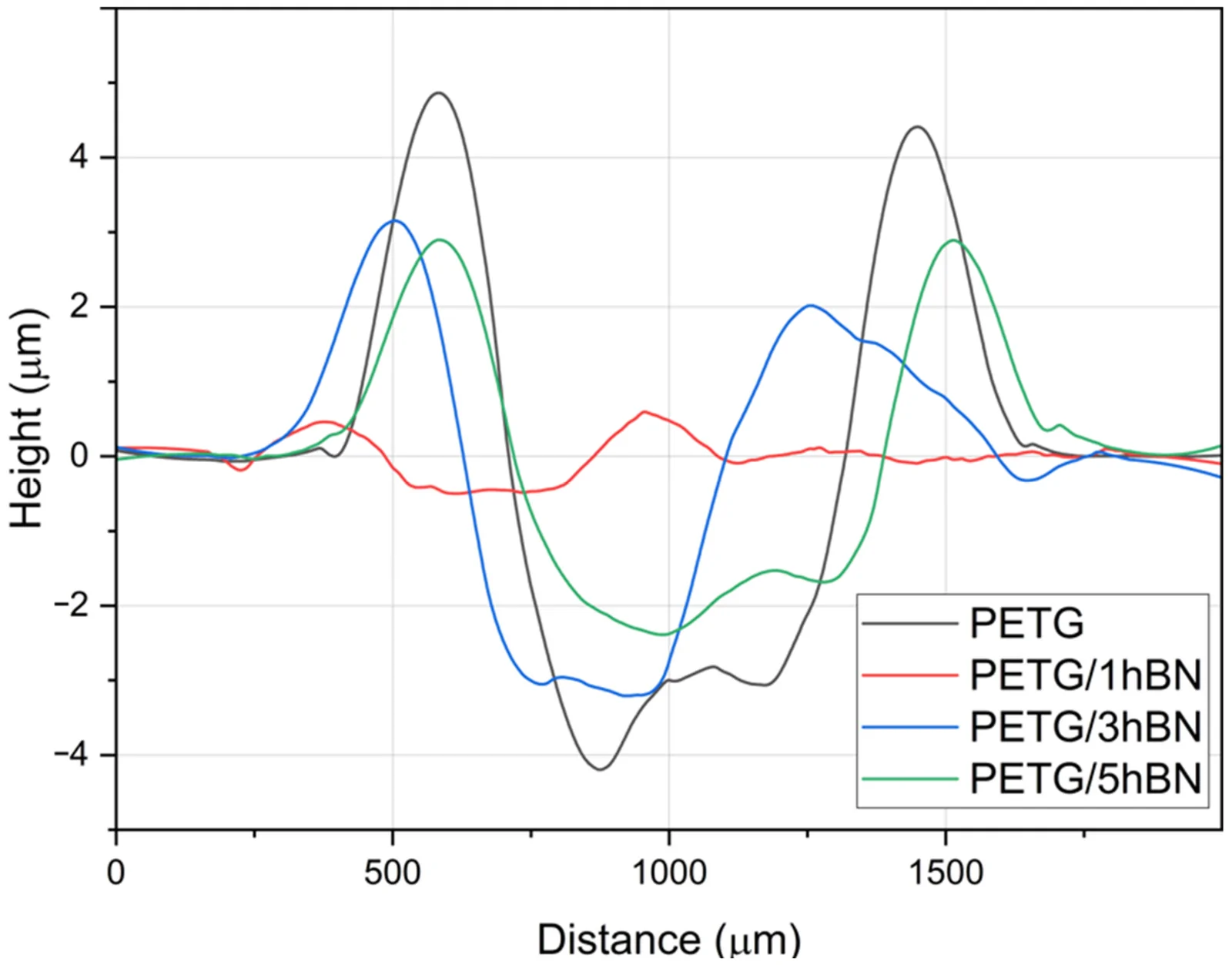
Two-dimensional profilometry cross-sections of the wear tracks of neat PETG and PETG/h-BN composites at 10 N normal load.

**Figure 15:**
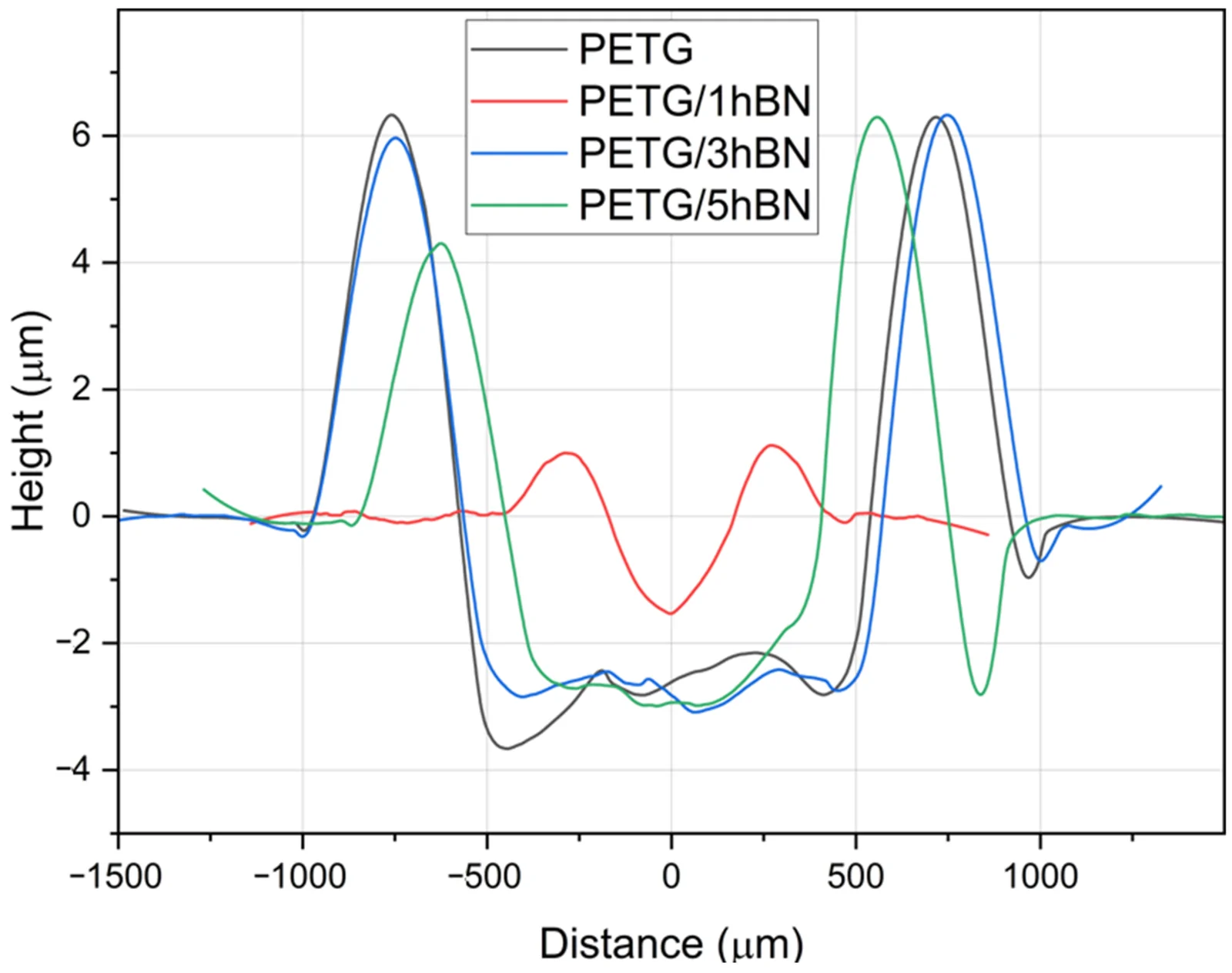
Two-dimensional profilometry cross-sections of the wear tracks of neat PETG and PETG/h-BN composites at 15 N normal load.

**Figure 16:**
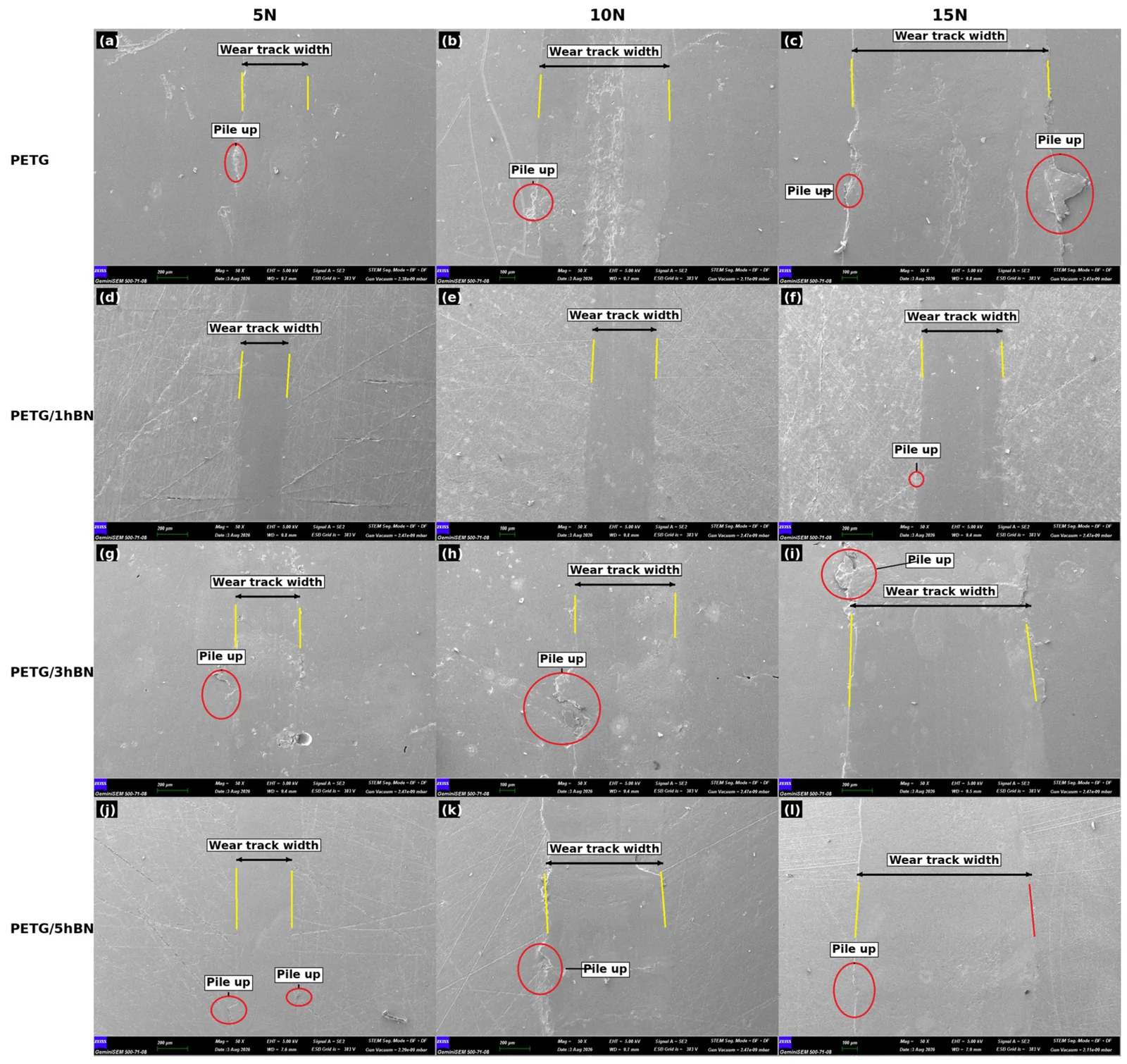
SEM micrographs of the wear tracks of neat PETG and PETG/h-BN composites at different normal loads: (**a**–**c**) neat PETG, (**d**–**f**) PETG/1hBN, (**g**–**i**) PETG/3hBN, and (**j**–**l**) PETG/5hBN, tested at 5, 10, and 15 N, respectively, at ×50 magnification. The wear track widths are indicated as between yellow lines, while the regions exhibiting pile-up are marked in red.

**Figure 17:**
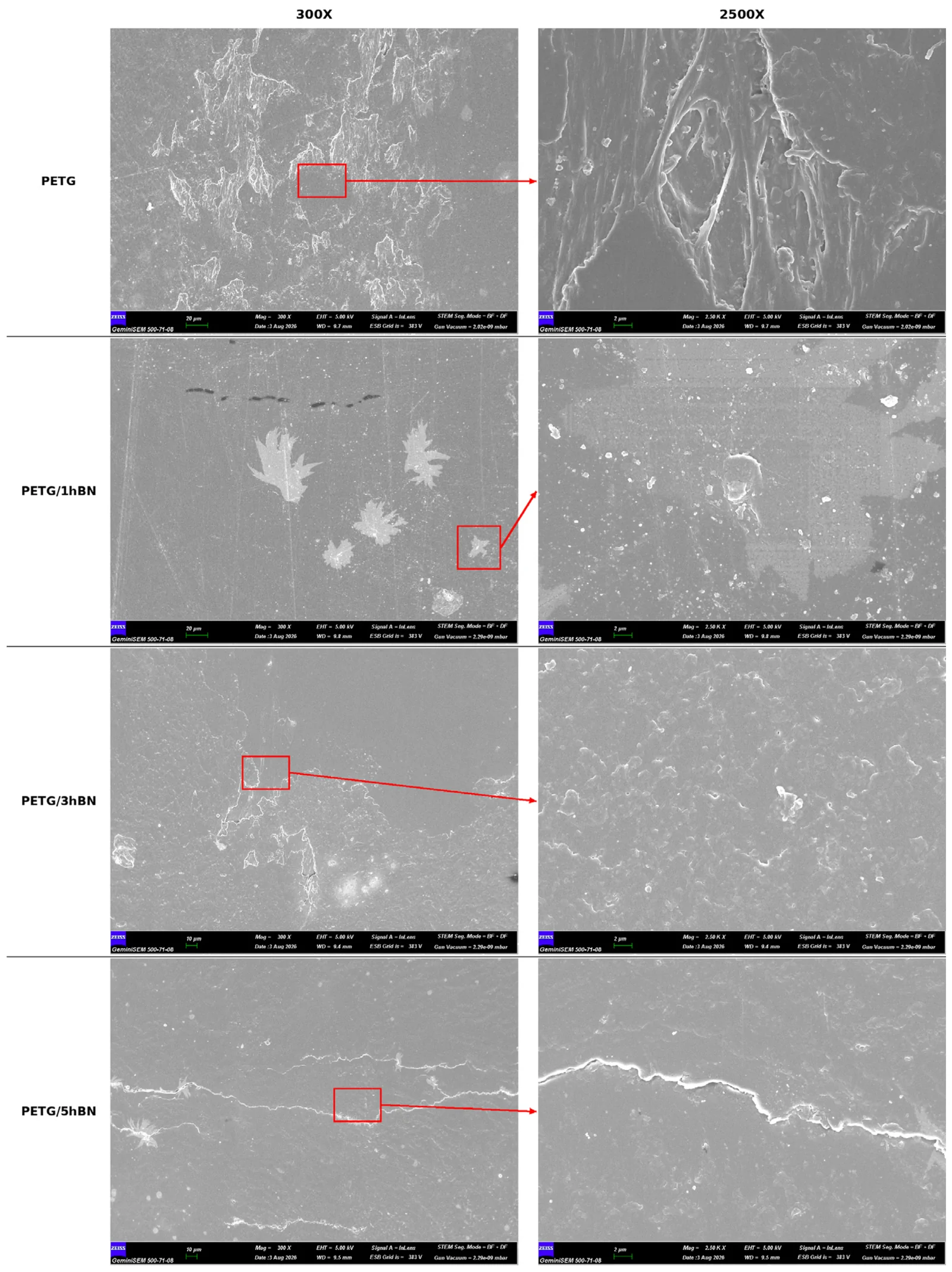
Comparative SEM images of neat PETG and h-BN-filled PETG composites at 10 N wear load, showing ×300 overview (**left**) and ×2500 magnified detail (**right**) of the marked region for each composition: neat PETG, PETG/1hBN, PETG/3hBN, PETG/5hBN. Red boxes and arrows indicate the zoomed-in region within each ×300 image.

**Figure 18:**
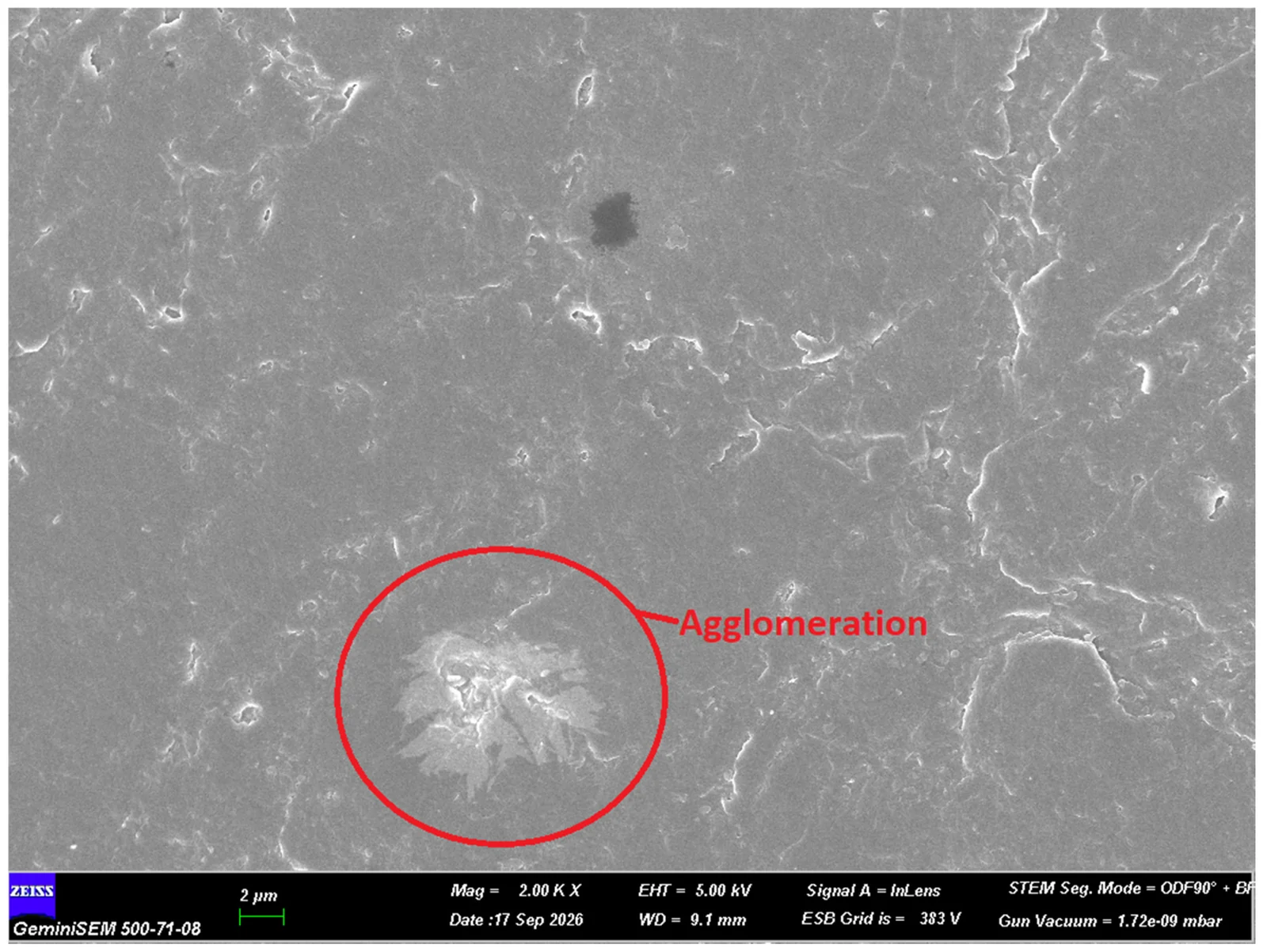
SEM image of the unworn PETG/5hBN composite surface showing a relatively large agglomerated h-BN region.

**Figure 19:**
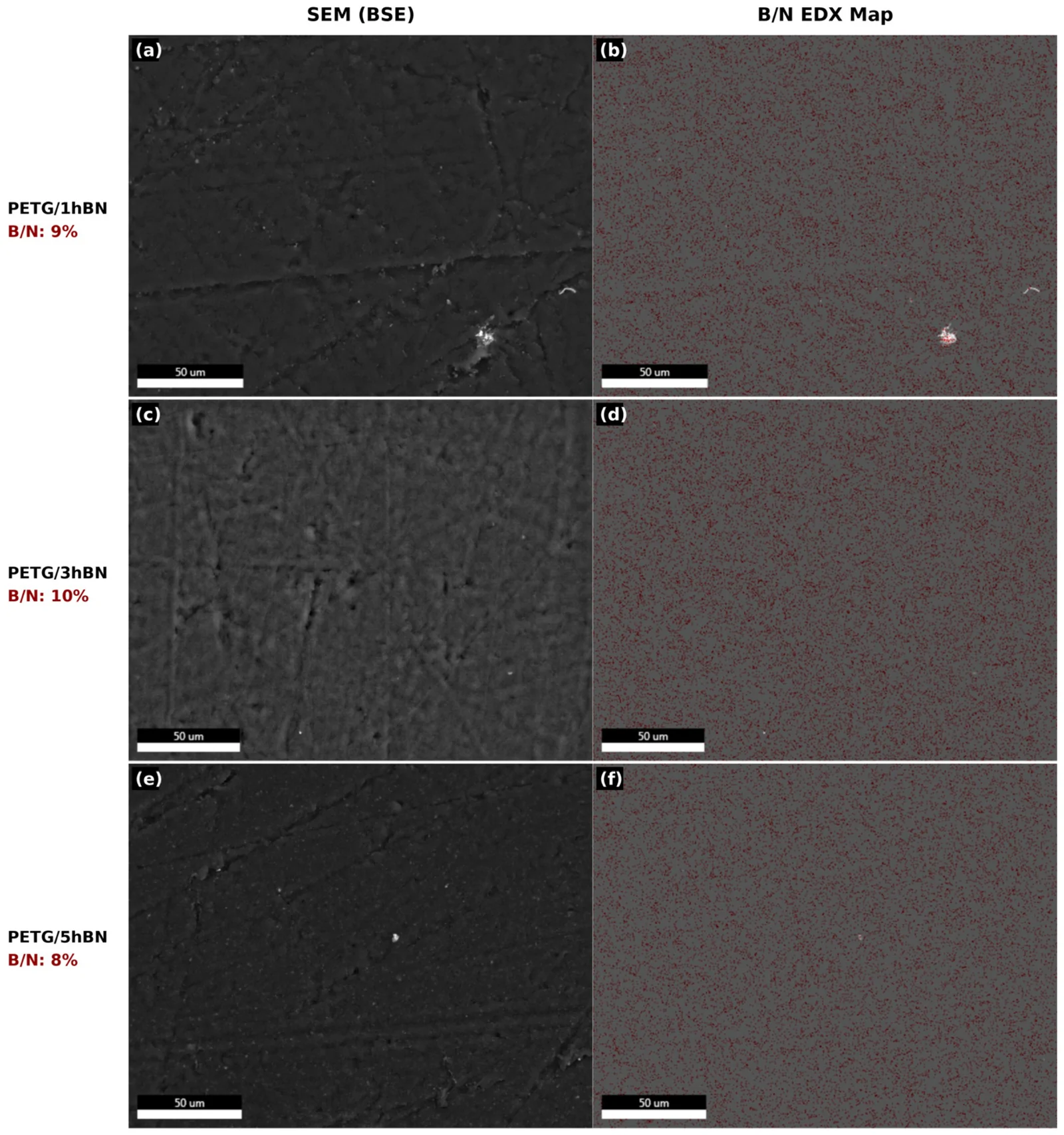
SEM (BSE) images and corresponding B/N EDX elemental maps of unworn (as-printed) PETG/h-BN composite surfaces: (**a**,**b**) PETG/1hBN; (**c**,**d**) PETG/3hBN; (**e**,**f**) PETG/5hBN. The percentage indicates the area fraction of the mapped region showing the B/N signal.

**Figure 20:**
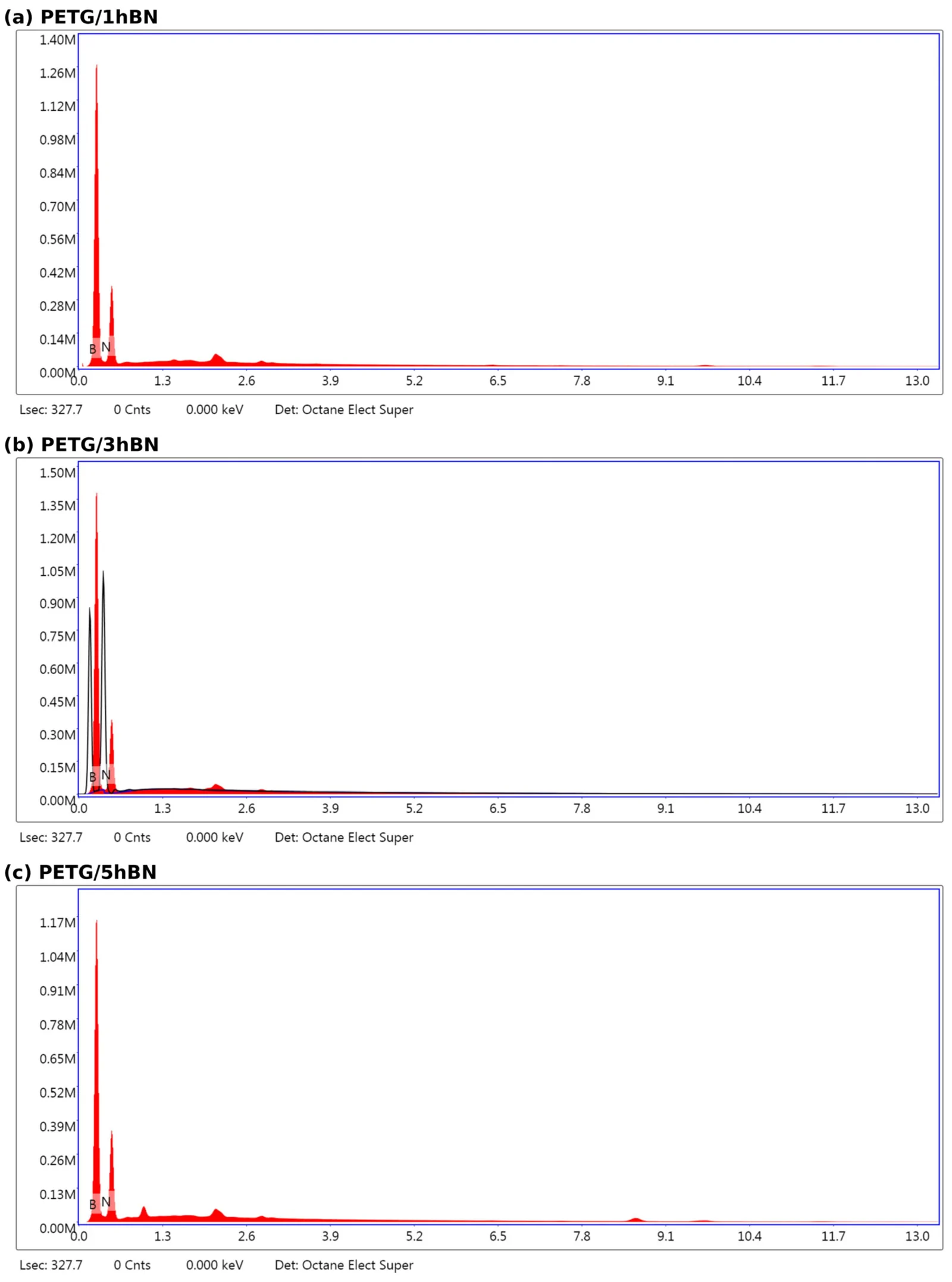
Representative EDX spectra (energy vs. counts) of the wear track surfaces of (**a**) PETG/1hBN, (**b**) PETG/3hBN, and (**c**) PETG/5hBN, showing the characteristic B and N peaks (20 kV accelerating voltage, 35.3° takeoff angle).

**Table 1:** FDM printing parameters.

Parameter	Value
Nozzle temperature (first layer)	255 °C
Nozzle temperature (other layers)	255 °C
Build plate temperature	70 °C
Maximum volumetric flow rate	12 mm^3^/s
Initial layer speed	50 mm/s
Initial layer infill speed	105 mm/s
Outer wall speed	200 mm/s
Inner wall speed	300 mm/s
Sparse infill speed	270 mm/s
Internal solid infill speed	250 mm/s
Vertical shell speed	80% of base speed
Top surface speed	200 mm/s
Overhang slow-down	Enabled

**Table 2:** Measured dimensions of FDM-printed specimens compared with the nominal CAD dimensions (30 × 30 × 5 mm); values are reported as mean ± standard deviation (*n* = 3 per composition).

Composition	X (mm)	Y (mm)	Z (mm)	Deviation X (%)	Deviation Y (%)	Deviation Z (%)
Neat. PETG	29.85 ± 0.03	29.77 ± 0.03	4.993 ± 0.006	−0.50	−0.77	−0.13
PETG/1hBN	29.82 ± 0.03	29.97 ± 0.03	5.027 ± 0.040	−0.60	−0.10	+0.53
PETG/3hBN	29.91 ± 0.02	29.94 ± 0.07	4.993 ± 0.006	−0.31	−0.19	−0.13
PETG/5hBN	29.79 ± 0.10	29.82 ± 0.0	5.010 ± 0.010	−0.71	−0.59	+0.20

**Table 3:** Tensile properties of neat PETG and PETG/h-BN composites.

Sample	Elastic Modulus (MPa)	UTS (MPa)	Elongation at Break(%)
Neat. PETG	1016.9	65.46	89.04
PETG/1hBN	1021.1	65.67	42.34
PETG/3hBN	809.1	47.28	24.45
PETG/5hBN	1175.3	51.85	30.03

**Table 4:** Compression properties of neat PETG and PETG/h-BN composites.

Sample	Elastic Modulus (MPa)	% Change	Compressive Yield Strength (MPa)	% Change
Neat. PETG	562.3	0	46.33	0
PETG/1hBN	569.4	+1.26	48.80	+5.33
PETG/3hBN	674.7	+19.99	42.26	−8.78
PETG/5hBN	1074.3	+91.05	45.03	−2.81

**Table 5:** Average coefficient of friction (COF) of neat PETG and PETG/h-BN composites as a function of normal load (mean ± SD).

Sample	COF at 5 N	COF at 10 N	COF at 15 N
Neat. PETG	0.489 ± 0.012	0.508 ± 0.015	0.525 ± 0.019
PETG/1hBN	0.264 ± 0.034	0.338 ± 0.016	0.313 ± 0.021
PETG/3hBN	0.402 ± 0.009	0.418 ± 0.017	0.474 ± 0.013
PETG/5hBN	0.460 ± 0.018	0.439 ± 0.004	0.535 ± 0.013

**Table 6:** Elemental composition (EDX, wt%) of the wear track surfaces of neat PETG and PETG/h-BN composites at 5, 10, and 15 N normal load.

Sample	Load (N)	C (wt%) ± std	O (wt%) ± std	B (wt%) ± std	N (wt%) ± std
Neat PETG	5	66.51	33.49	-	-
Neat PETG	10	66.48	33.52	-	-
Neat PETG	15	66.92	33.08	-	-
PETG/1hBN	5	67.290 ± 0.452	28.697 ± 0.140	2.890 ± 0.082	1.123 ± 0.379
PETG/1hBN	10	66.500 ± 0.502	29.103 ± 0.280	3.023 ± 0.081	1.373 ± 0.363
PETG/1hBN	15	67.013 ± 0.784	29.267 ± 0.074	2.703 ± 0.219	1.017 ± 0.638
PETG/3hBN	5	64.923 ± 0.991	29.650 ± 0.317	3.070 ± 0.079	2.357 ± 0.732
PETG/3hBN	10	65.867 ± 0.542	29.847 ± 0.333	2.943 ± 0.042	1.343 ± 0.506
PETG/3hBN	15	64.713 ± 1.774	29.333 ± 0.371	3.380 ± 0.135	2.567 ± 1.284
PETG/5hBN	5	63.523 ± 0.994	28.660 ± 0.589	4.067 ± 0.240	3.753 ± 0.644
PETG/5hBN	10	63.927 ± 0.772	30.103 ± 0.715	3.220 ± 0.197	2.747 ± 0.240
PETG/5hBN	15	62.543 ± 2.067	29.083 ± 1.666	4.193 ± 0.239	4.180 ± 0.641

**Table 7:** Cost comparison for an equivalent component volume: PETG/1hBN versus bronze.

Cost Component	PETG/1hBN	Bronze
Material density (g/cm^3^)	1.29 *	8.80
Material required for equivalent volume (kg)	1.00	6.82
PETG cost (€/kg)	2.76	-
h-BN cost (€/kg)	4.73	-
Bronze cost (€/kg)	-	13.41
PETG/h-BN material cost (€)	7.46	-
Bronze material cost (€)	-	91.46
Twin-screw compounding (€/kg)	3.58	-
Filament production (€/kg)	2.43	-
FDM processing (€/kg)	17.88	-
Machining cost (€)	-	137.18 **
Total production cost (€)	31.34	228.64
Relative cost	1.00×	7.30×

* PETG density used for the equivalent-volume calculation. ** Machining cost was estimated as 1.5 × bronze material cost, as an adopted cost assumption.

## Data Availability

The raw data supporting the conclusions of this article will be made available by the authors on request.

## Abbreviations

The following abbreviations are used in this manuscript:

ABS	Acrylonitrile butadiene styrene
ASTM	American Society for Testing and Materials
COF	Coefficient of friction
EDX	Energy-dispersive X-ray spectroscopy
FDM	Fused deposition modeling
h-BN	Hexagonal boron nitride
PA	Polyamide
PAEK	Poly(aryl ether ketone)
PEEK	Poly(ether ether ketone)
PETG	Polyethylene terephthalate glycol
PLA	Polylactic acid
PTFE	Polytetrafluoroethylene
Ra	Arithmetic mean roughness
Rku	Kurtosis
Rp	Maximum peak height
Rq	Root-mean-square roughness
Rsk	Skewness
Rt	Total profile height
Rv	Maximum valley depth
Rz	Mean peak-to-valley height
SEM	Scanning electron microscopy
UTS	Ultimate tensile strength

## References

[1] Wickramasinghe S., Do T., Tran P. (2020). FDM-based 3D printing of polymer and associated composite: A review on mechanical properties, defects and treatments. Polymers.

[2] Gordelier T.J., Thies P.R., Turner L., Johanning L. (2019). Optimising the FDM additive manufacturing process to achieve maximum tensile strength: A state-of-the-art review. Rapid Prototyp. J..

[3] Jamal M.A., Shah O.R., Ghafoor U., Qureshi Y., Bhutta M.R. (2024). Additive Manufacturing of Continuous Fiber-Reinforced Polymer Composites Via Fused Deposition Modelling: A Comprehensive Review. Polymers.

[4] Mohamed O.A., Masood S.H., Bhowmik J.L. (2017). Investigation on the tribological behavior and wear mechanism of parts processed by fused deposition additive manufacturing process. J. Manuf. Process..

[5] Dangnan F., Espejo C., Liskiewicz T., Gester M., Neville A. (2020). Friction and wear of additive manufactured polymers in dry contact. J. Manuf. Process..

[6] Amirruddin M.S., Ismail K.I., Yap T.C. (2022). Effect of layer thickness and raster angle on the tribological behavior of 3D printed materials. Mater. Today Proc..

[7] Rangisetty S., Peel L.D. The Effect of Infill Patterns and Annealing on Mechanical Properties of Additively Manufactured Thermoplastic Composites. Proceedings of the ASME 2017 Conference on Smart Materials, Adaptive Structures and Intelligent Systems (SMASIS2017).

[8] Cuiffo M.A., Snyder J., Elliott A.M., Romero N., Kannan S., Halada G.P. (2017). Impact of the fused deposition (FDM) printing process on polylactic acid (PLA) chemistry and structure. Appl. Sci..

[9] Kumaresan R., Kanny K. (2025). Advanced RSM-Driven Optimisation for Enhancing the Mechanical Performance of FDM-Printed PETG: A Correlated Microstructural and Mechanical Property Investigation. Polymers.

[10] Hsueh M.-H., Lai C.-J., Wang S.-H., Zeng Y.-S., Hsieh C.-H., Pan C.-Y., Huang W.-C. (2021). Effect of Printing Parameters on the Thermal and Mechanical Properties of 3D-Printed PLA and PETG, Using Fused Deposition Modeling. Polymers.

[11] Patel K.S., Shah D.B., Joshi S.J., Aldawood F.K., Kchaou M. (2024). Effect of process parameters on the mechanical performance of FDM printed carbon fiber reinforced PETG. J. Mater. Res. Technol..

[12] Vaught L.O., Polycarpou A.A. (2023). Investigating the effect of fused deposition modelling on the tribology of PETG thermoplastic. Wear.

[13] Kichloo A.F., Raina A., Haq M.I.U., Wani M.S. (2022). Impact of Carbon Fiber Reinforcement on Mechanical and Tribological Behavior of 3D-Printed Polyethylene Terephthalate Glycol Polymer Composites—An Experimental Investigation. J. Mater. Eng. Perform..

[14] Kumar K.S., Soundararajan R., Shanthosh G., Saravanakumar P., Ratteesh M. (2021). Augmenting effect of infill density and annealing on mechanical properties of PETG and CFPETG composites fabricated by FDM. Mater. Today Proc..

[15] Mahesh V., George J.P., Mahesh V., Chakraborthy H., Mukunda S., Ponnusami S.A. (2024). Dry-sliding wear properties of 3D printed PETG/SCF/OMMT nanocomposites: Experimentation and model predictions using artificial neural network. J. Reinf. Plast. Compos..

[16] Bedi S.S., Mallesha V. (2026). Investigating tribological performance in 3D-printed PETG/graphene composites of varying composition. J. Reinf. Plast. Compos..

[17] Raja S., Jayalakshmi M., Rusho M.A., Selvaraj V.K., Subramanian J., Yishak S., Kumar T.A. (2024). Fused deposition modeling process parameter optimization on the development of graphene enhanced polyethylene terephthalate glycol. Sci. Rep..

[18] Hanon M.M., Marczis R., Zsidai L. (2021). Impact of 3D-Printing Structure on the Tribological Properties of Polymers. Ind. Lubr. Tribol..

[19] Bahadur S., Gong D. (1992). The action of fillers in the modification of the tribological behavior of polymers. Wear.

[20] Schwartz C.J., Bahadur S. (2000). Studies on the tribological behavior and transfer film–counterface bond strength for polyphenylene sulfide filled with nanoscale alumina particles. Wear.

[21] Blanchet T.A., Kennedy F.E. (1992). Sliding wear mechanism of polytetrafluoroethylene (PTFE) and PTFE composites. Wear.

[22] Ye J., Khare H.S., Burris D.L. (2013). Transfer film evolution and its role in promoting ultra-low wear of a PTFE nanocomposite. Wear.

[23] Khare H.S., Burris D.L. (2016). A Review of Transfer Films and Their Role in Ultra-Low-Wear Sliding of Polymers. Lubricants.

[24] Chkhartishvili L., Tabatadze G., Nackebia D., Bzhalava T., Kalandadze I. (2016). Hexagonal boron nitride as a solid lubricant additive (an overview). Nano Stud..

[25] Wu X., Han Q. (2020). Thermal conductivity of monolayer hexagonal boron nitride: From defective to amorphous. Comput. Mater. Sci..

[26] Zhang W., Liang Y., Yue D., Feng Y. (2024). Hexagonal boron nitride nanosheets: Fabrication, thermal properties and application in polymers. High Perform. Polym..

[27] Kodali V., Roberts J.R., Glassford E., Gill R., Friend S., Dunn K.L., Erdely A. (2022). Understanding toxicity associated with boron nitride nanotubes: Review of toxicity studies, exposure assessment at manufacturing facilities, and read-across. J. Mater. Res..

[28] He J., Zhang X., Liu L., Wang Y., Liu R., Li M., Gao F. (2023). Acute and Subacute Toxicity Evaluation of Erythrocyte Membrane-Coated Boron Nitride Nanoparticles. J. Funct. Biomater..

[29] Ramteke S., Chelladurai H. (2020). Examining the role of hexagonal boron nitride nanoparticles as an additive in the lubricating oil and studying its application. Proc. Inst. Mech. Eng. Part N J. Nanomater. Nanoeng. Nanosyst..

[30] Kimura Y., Wakabayashi T., Okada K., Wada T., Nishikawa H. (1999). Boron nitride as a lubricant additive. Wear.

[31] Pawlak Z., Kaldonski T., Pai R., Bayraktar E., Oloyede A. (2009). A comparative study on the tribological behavior of hexagonal boron nitride (hBN) as lubricating microparticles—An additive in porous sliding bearings for a car clutch. Wear.

[32] Joshi M.D., Goyal A., Patil S.M., Goyal R.K. (2017). Tribological and thermal properties of hexagonal boron nitride filled high-performance polymer nanocomposites. J. Appl. Polym. Sci..

[33] De Oliveira P.R., Kunneparambil Sukumaran A., Benedetti L., John D., Stephens K., Chu S.-H., Park C., Agarwal A. (2023). Novel polyimide-hexagonal boron nitride nanocomposites for synergistic improvement in tribological and radiation shielding properties. Tribol. Int..

[34] Mistry M., Randhawa K.S. (2020). Investigations of the influence of hexagonal boron nitride particulates on mechanical & tribological properties of PA66. J. Phys. Conf. Ser..

[35] Vikram K., Pramanik S., Bhaumik S. (2024). Effect of hexagonal boron nitride on structural, mechanical, and tribological behavior of polyamide 6/glass fibers (5 wt%) hybrid nanocomposites. Iran. Polym. J..

[36] Randhawa K.S., Patel A.D. (2020). Enhancing tribo-mechanical properties and thermal stability of nylon 6 by hexagonal boron nitride fillers. e-Polymers.

[37] Panda J.N., Bijwe J., Pandey R.K. (2019). Optimization of the amount of short glass fibers for superior wear performance of PAEK composites. Compos. Part A Appl. Sci. Manuf..

[38] Panda J.N., Bijwe J., Pandey R.K. (2019). Role of micro and nano-particles of hBN as a secondary solid lubricant for improving tribo-potential of PAEK composite. Tribol. Int..

[39] Chen J., Chen B., Li J., Tong X., Zhao H., Wang L. (2017). Enhancement of mechanical and wear resistance performance in hexagonal boron nitride-reinforced epoxy nanocomposites. Polym. Int..

[40] Wang S., Li Q., Zhang S., Pan L. (2013). Tribological behavior of poly(phenyl p-hydroxybenzoate)/polytetrafluoroethylene composites filled with hexagonal boron nitride under dry sliding condition. Mater. Des..

[41] He G., Li Y., Wu L., Wang Y., Liu M., Yuan J., Men X. (2021). Synergy of core-shell Cu@rGO hybrids for significantly improved thermal and tribological properties of polyimide composites. Tribol. Int..

[42] Wan C., Zhan S., Jia D., Yang T., Chen H., Yao C., Duan H. (2022). Tribological behavior of nanographite/polyimide composite under drying sliding condition. Wear.

[43] Song J., Dai Z., Li J., Tong X., Zhao H. (2018). Polydopamine-decorated boron nitride as nano-reinforcing fillers for epoxy resin with enhanced thermomechanical and tribological properties. Mater. Res. Express.

[44] Lin Z., McNamara A., Liu Y., Moon K.S., Wong C.P. (2014). Exfoliated Hexagonal Boron Nitride-Based Polymer Nanocomposite with Enhanced Thermal Conductivity for Electronic Encapsulation. Compos. Sci. Technol..

[45] Cho D.H., Kim J.S., Kwon S.H., Lee C., Lee Y.Z. (2013). Evaluation of hexagonal boron nitride nano-sheets as a lubricant additive in water. Wear.

[46] Ralls A.M., Monette Z., Kasar A.K., Menezes P.L. (2024). Enhancing Tribological Performance of Self-Lubricating Composite via Hybrid 3D Printing and In Situ Spraying. Materials.

[47] (2014). Standard Test Method for Tensile Properties of Plastics.

[48] (2015). Standard Test Method for Compressive Properties of Rigid Plastics.

[49] (2022). Standard Test Method for Linearly Reciprocating Ball-on-Flat Sliding Wear.

[50] Javorova J.G. (2020). Ball-on-flat reciprocating test to evaluate dry sliding wear behaviour of reinforced polymer composites. IOP Conf. Ser. Mater. Sci. Eng..

[51] Iliuță V., Rîpă M., Preda A., Andrei G. (2015). Friction and Wear Behavior of Moglice Polymer Composite through Dry Sliding Ball-on-Flat Reciprocating Test. Appl. Mech. Mater..

